# The NAG Sensor NagC Regulates LEE Gene Expression and Contributes to Gut Colonization by *Escherichia coli* O157:H7

**DOI:** 10.3389/fcimb.2017.00134

**Published:** 2017-04-24

**Authors:** Guillaume Le Bihan, Jean-Félix Sicard, Philippe Garneau, Annick Bernalier-Donadille, Alain P. Gobert, Annie Garrivier, Christine Martin, Anthony G. Hay, Francis Beaudry, Josée Harel, Grégory Jubelin

**Affiliations:** ^1^Faculté de Médecine Vétérinaire, Centre de Recherche en Infectiologie Porcine et Aviaire, Université de MontréalSaint-Hyacinthe, QC, Canada; ^2^INRA, Université Clermont Auvergne, MEDISClermont-Ferrand, France; ^3^Department of Microbiology, Cornell UniversityIthaca, NY, USA; ^4^Groupe de Recherche en Pharmacologie Animal du Québec, Département de Biomédecine Vétérinaire, Faculté de Médecine Vétérinaire, Université de MontréalSaint-Hyacinthe, QC, Canada

**Keywords:** NagC, LEE, EHEC, N-acetylglucosamine (or eventually NAG), N-acetylneuraminic acid (or eventually NANA)

## Abstract

Enterohemorrhagic *Escherichia coli* (EHEC) O157:H7 are human pathogens responsible for bloody diarrhea and renal failures. EHEC employ a type 3 secretion system to attach directly to the human colonic epithelium. This structure is encoded by the locus of enterocyte effacement (LEE) whose expression is regulated in response to specific nutrients. In this study, we show that the mucin-derived sugars N-acetylglucosamine (NAG) and N-acetylneuraminic acid (NANA) inhibit EHEC adhesion to epithelial cells through down-regulation of LEE expression. The effect of NAG and NANA is dependent on NagC, a transcriptional repressor of the NAG catabolism in *E. coli*. We show that NagC is an activator of the LEE1 operon and a critical regulator for the colonization of mice intestine by EHEC. Finally, we demonstrate that NAG and NANA as well as the metabolic activity of *Bacteroides thetaiotaomicron* affect the *in vivo* fitness of EHEC in a NagC-dependent manner. This study highlights the role of NagC in coordinating metabolism and LEE expression in EHEC and in promoting EHEC colonization *in vivo*.

## Introduction

*Escherichia coli* O157:H7 are human foodborne pathogens responsible for outbreaks mostly in developed countries. Infections by EHEC occur following ingestion of contaminated food and provoke symptoms ranging from watery or bloody diarrhea to hemolytic and uremic syndrome (HUS). A range of virulence factors are involved in EHEC O157:H7 pathogenicity including the Shiga-toxin which is associated with development of HUS, and the T3SS which enables the pathogen to attach to the intestinal epithelium and cause diarrhea (Kaper et al., 2004).

T3SS-encoding genes are gathered into the locus of enterocyte effacement (LEE) that is composed of five operons (LEE1 to LEE5) which encode for structural proteins, regulators, chaperones and effectors that are secreted into the host cells (Kaper et al., 2004). The first gene of the LEE, *ler*, encodes an activator that regulates the five major LEE operons. Expression of *ler* is controlled by several regulators in response to intestinal metabolites, such as bacterial waste products (Nakanishi et al., 2009), quorum-sensing molecules (Sircili et al., 2004), hormones (Walters and Sperandio, 2006), biotin (Yang et al., 2015), fucose (Pacheco et al., 2012), and ethanolamine (Kendall et al., 2012).

During its infectious cycle, EHEC O157:H7 encounters a large amount of mucin-derived sugars (Fabich et al., 2008; Bertin et al., 2013). Mucin is part of the mucous layer covering the intestinal epithelium and is heavily O-glycosylated. The mucous layer is a physical barrier that limits contact between bacteria and host epithelial cells (McGuckin et al., 2011). By producing specific glycosidases, several species of the gut microbiota release sugars from O-glycans into the intestinal lumen (Bertin et al., 2013; Ng et al., 2013; Elhenawy et al., 2014). Released mucin sugars, including N-acetylglucosamine (NAG), N-acetylneuraminic acid (NANA), galactose, fucose, mannose and N-acetylgalactosamine, represent an important reservoir of nutrients that promotes the growth of commensal and pathogenic bacteria including *E. coli* (Fabich et al., 2008; Bertin et al., 2013; Conway and Cohen, 2015). *Escherichia coli* and more particularly EHEC O157:H7 are able to concomitantly metabolize *in vitro* up to nine mucin sugars at a time, and preferentially use NAG and galactose (Fabich et al., 2008; Bertin et al., 2013; Conway and Cohen, 2015).

Genes involved in the catabolism of sugars are often regulated by proteins responding to the presence of their cognate sugar. For example, the regulator NagC, known as a repressor of NAG and galactose catabolism, is a NAG-6 phosphate (NAG-6P) sensing protein, NAG-6P being produced during the catabolism of NAG and NANA (Plumbridge, 1991; El Qaidi et al., 2009). When NAG-6P concentrations are low, NagC acts as a DNA binding protein, an activity that is lost with high intracellular NAG-6P concentration (Plumbridge and Kolb, 1991; Sohanpal et al., 2004). In addition to the role they play as nutrients, some mucin sugars can act as regulatory signals that influence bacterial colonization and adherence to cells (Sohanpal et al., 2004; Barnhart et al., 2006; Pacheco et al., 2012).

Previously, we have shown that EHEC O157:H7 respond to the metabolic activity of the human gut microbiota by activating the expression of genes required for NANA utilization and by down-regulating the expression of the LEE genes (Le Bihan et al., 2015). In this study, the effect of NANA and NAG on the adhesion phenotype of EHEC O157:H7 was examined. We found that NANA and NAG are inhibitors of EHEC O157:H7 adhesion to epithelial cells. We demonstrated that NANA and NAG reduce the expression of the five LEE operons in a NagC-dependent way. Mutation in *nagC* diminished the expression of LEE genes. In addition, NagC was shown to bind directly to the LEE1 promoter region, thereby could influence expression of *ler* gene, which encodes the LEE master regulator. We also show that NagC promotes EHEC colonization of mouse intestine. Further, we demonstrate that exogenous addition of NAG into the intestine or gavage with the mucin degrading commensal *Bacteroides thetaiotaomicron* modulates the fitness of EHEC *in vivo* in a NagC-dependent manner. Taken together, our data indicate that NagC coordinates the catabolism of mucus-derived sugars and T3SS production, and promotes EHEC intestinal colonization.

## Materials and methods

### Bacteria, mutagenesis, and growth conditions

Strains and plasmids are listed in Table EV1. The EHEC O157:H7 strain EDL933 (O'Brien et al., 1983) was used in this study. *B. thetaiotamicron* strain VPI-5482 was grown anaerobically at 37°C in a complex medium containing clarified rumen fluid (Leedle and Hespell, 1980). The medium was prepared, dispensed and inoculated by using strictly anaerobic techniques in Balch tubes. The EDL933 Δ*nagC* and Δ*nanR* mutants were generated by allelic exchange using a suicide vector as described in EV Methods. When required, the growth medium was supplemented with kanamycin (25 mg/ml), ampicillin (50 mg/ml), NANA (0.1 mM or 1 mM), or NAG (0.1 mM or 1 mM) (Sigma Aldrich).

### Beta-galactosidase assays

The entire intergenic region between *ler* (LEE1) and *espG* (bp −1,225 to +19) containing two *ler* promoters (Sperandio et al., 2002; Porter et al., 2005) was inserted upstream of *lacZ* in pRS551 (see EV Methods). The resulting plasmid pGLB was introduced into EDL933 or its isogenic mutants. After growth in DMEM with or without NANA or NAG until OD_600_ of 0.6, β-galactosidase assays were performed as described previously (Miller, 1972). Student *t*-tests were performed to determine statistical significance.

### Quantitative real time PCR (qRT-PCR)

Bacteria were harvested at OD_600_ of 0.6 and RNA was extracted as previously described (Le Bihan et al., 2015). cDNAs were synthesized from 10 μg RNA using reverse transcriptase. The concentration of cDNA samples was then adjusted to 25 ng/μL. A standard curve was performed for genes of interest to determine the copy number of targeted transcripts in 50 ng of cDNA. Primers used in this study are listed in Table EV2. Results are presented as the ratios between the cDNA copy number of the gene of interest and the cDNA copy number of the housekeeping gene. Student *t*-tests were performed to calculate *p*-values.

### Western blotting

Western blot analyses were performed with slight modifications to those previously described (Chekabab et al., 2014). Culture supernatants (8 ml) were harvested and supplemented with 1 μg Bovine Serum Albumin (BSA). Proteins were precipitated overnight at 4°C using 10% trichloroacetic acid and sodium deoxycholate, pelleted by centrifugation, washed with acetone, resuspended in SDS sample buffer and boiled. Proteins were then run on 14% SDS-PAGE gels and transferred to nitrocellulose membranes. Protein transfer including BSA was assessed using Ponceau S dye. The EspB protein was revealed using a rabbit EspB specific polyclonal antiserum (1:2,000) and a goat anti-rabbit HRP-linked secondary antibody (Bio-Rad Laboratories, Hercules, CA).

### Cell culture and bacterial infections

Epithelial cell lines HeLa, HCT-8, and HCT-116 were maintained in MEM with 10% FBS, 100 U/mL penicillin and 100 mg/mL streptomycin at 37°C under 5% CO_2_(Branchu et al., 2014). Cells were seeded into 6-well plates (5 × 10^5^ cells/well) and grown for 24 h without antibiotics. Bacteria were pre-grown in DMEM in the presence or absence of NANA or NAG before the infection assays. Cells were then washed and infected with bacteria with an MOI of 10 for 90 min, in the presence or absence of NANA or NAG. Following infection, cells were washed 3 times with DPBS, trypsinized for 5 min at 37°C, pelleted by a low-speed centrifugation (100 *g*; 3 min). The pellet was washed once to ensure the plating of adherent bacteria only. Results are presented as the percentage of adherent bacteria as compared to the wild-type strain incubated without NANA or NAG. The EDL933 Δ*escN* strain that does not produce the T3SS (Deng et al., 2004) was used as a negative control. Student *t*-tests were performed to determine the significance.

### Electrophoretic mobility shift assays (EMSA)

The EMSA was adapted from previous report (Chekabab et al., 2014). The EMSA reaction mix consisted of purified NagC at the desired concentration (0.5–2.5 μM), 50 nM of 5′ 6-FAM labeled pLEE1 probe, 0.1 mg/mL calf thymus DNA and 0.1 mg/mL BSA in EMSA buffer (50 mM NaCl, 20 mM Tris, pH 7.4, 0.02% v/v sodium azide). Reactions were incubated for 30 min at 25°C, and then loaded onto a 12% native polyacrylamide gel running at 120 V in 1x TBE buffer. The forward primer contained a 5′ 6-FAM fluorescein tag. Competitive EMSA assay was done with 50 nM of 6-FAM labeled pLEE1 probe and unlabeled probes corresponding to P_LEE1_, P_kan_ (negative control) or P_*nagB*−*nagE*_ (positive control). The ratio “cold probe/labeled probe” was 10/1.

### DNAse footprinting

DNase I footprinting of free DNA and DNA–protein complexes was performed as described (El Qaidi et al., 2009; Graveline et al., 2011). The DNA fragment corresponding to the *ler* regulatory region (259 bp) was amplified using primers listed in Table EV2, alternately end labeled with ^32^P (140,000 cpm, 0.6 nM). Each end-labeled amplicon was subsequently incubated in a total volume of 80 μl in binding buffer (25 mM Hepes (pH 8.0), 100 mM K glutamate (pH 8.0), 0.5 mg/ml BSA). After incubation with purified NagC for 10 min at room temperature, 2 μl of DNase I (1.3 U/ml; New England BioLabs) containing 5 mM CaCl_2_ and 25 mM MgCl_2_ was added for 5 min. The reaction was stopped by the addition of 150 μL of phenol/chloroform/isoamyl alcohol and 350 μl of stop buffer (0.5 M Na acetate pH 5.0, 2.5 mM EDTA, 10 μg/ml Salmon sperm DNA) to each sample. DNA fragments were precipitated in ethanol, and amounts with equivalent cpm (5.10^4^) from each reaction were loaded onto 6% polyacrylamide–7 M urea gels. Maxam-Gilbert A+G reactions were carried out on the appropriate 32 P-labeled DNA fragments, and the products loaded alongside the DNase I footprinting reaction mixtures. The gels were dried and analyzed by autoradiography. A control footprinting experiment realized with *nagE-nagB* regulatory region and with NagC (Figure EV5).

### Mice infection

BALB/c mice were purchased from Janvier Labs (Le-Genest-St-Isle, France). Sets of 5 mice aged 5 weeks were given drinking water containing streptomycin sulfate (5 g/l) throughout the experiment. On day 1 following the addition of streptomycin, each mouse was infected intragastrically with 100 μl of a mix containing 10^7^ each of EDL933 Sm^R^ and EDL933 Sm^R^ Δ*nagC* strains. Mice treated with *B. thetaiotaomicron* were gavaged daily with 5 × 10^9^ cells of *B. thetaiotaomicron* strain VPI-5482, starting from day 1 before EHEC infection to day 7. At indicated time points, fecal or tissue samples were collected, homogenized in PBS and subsequently diluted before plating on LB-streptomycin agar plates and LB-streptomycin-kanamycin agar plates. Output ratios were calculated for each time point and competitive indices were obtained by dividing the output ratio by the input ratio. A One-way ANOVA was performed to determine the significance. For NANA and NAG quantification in intestinal contents, see EV Methods.

### Ethics statement

All animal experiments were reviewed and approved by the Auvergne Committee for Animal Experimentation (C2E2A). All procedures were carried out according to the European directives for the protection of animals used for scientific purposes, 2010/63/EU, and to the guidelines of the local ethics committee.

## Results

### NANA and NAG inhibit EHEC O157:H7 adhesion to epithelial cells by repressing LEE genes

**Figure 1:**
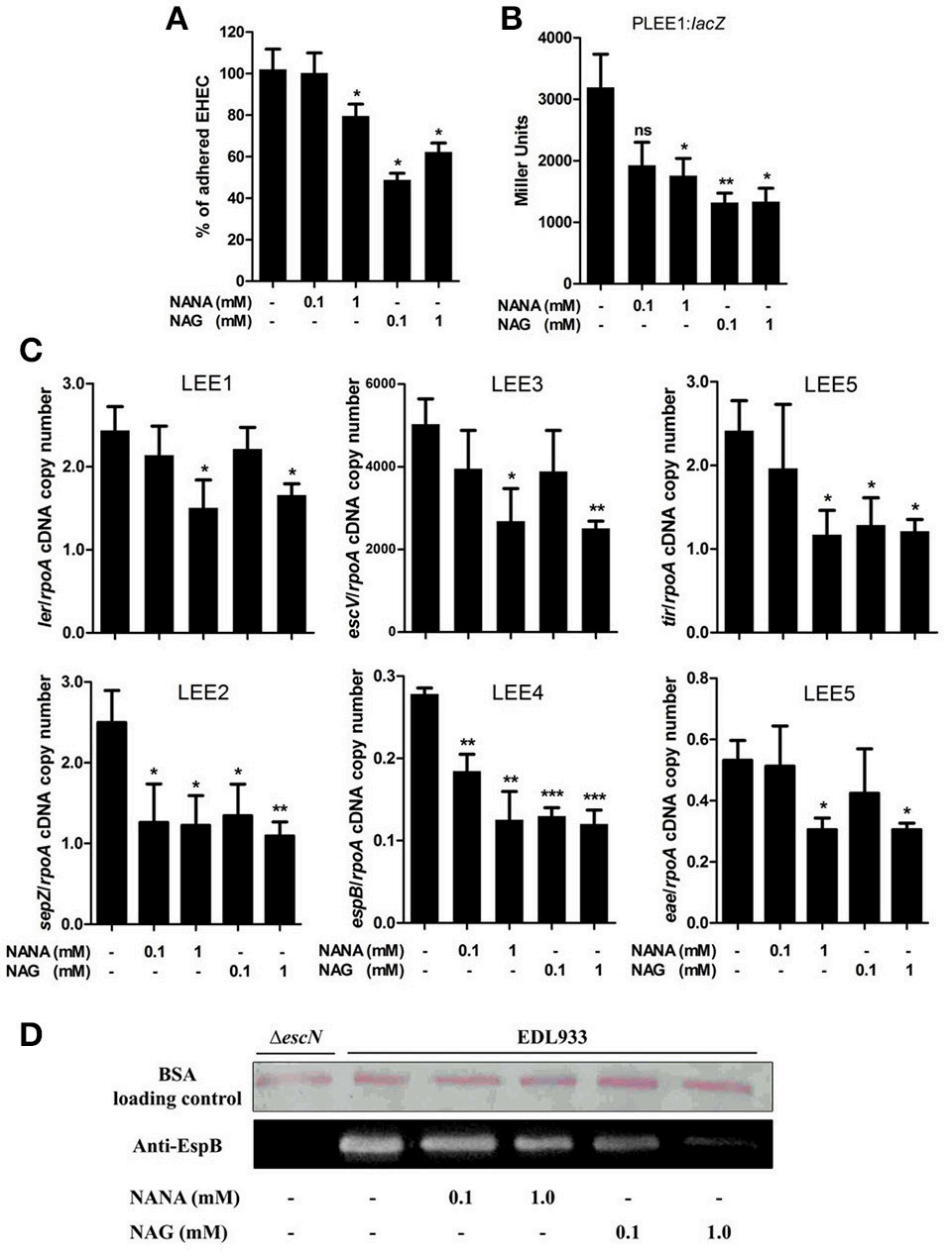
**NANA and NAG regulate EDL933 adhesion and LEE expression. (A)** HeLa cells were co-incubated with EDL933 in DMEM with or without NANA or NAG for 90 min. Bacteria adhered to HeLa cells were harvested and counted on agar plates. Results are presented as the percentage of adhesion as compared to the wild type strain incubated without NANA or NAG. **(B)** β-galactosidase assays using the P_LEE1_:*lacZ* transcriptional fusion integrated into EDL933. EDL933 was grown in DMEM with or without NANA or NAG 0.1 or 1 mM and cells were harvested at OD_600_ = 0.6. Results are presented as Miller Units. **(C)** qRT-PCR measurement of LEE gene expression in DMEM with or without NANA or NAG. Results are shown as the ratio copy number of the LEE transcripts/copy number of *rpoA* transcripts. **(D)** Western blot analysis of the EspB secretion by EDL933 grown in DMEM with or without NANA or NAG. BSA was used as a loading control. *n* ≥ 3, ns for non-significant, ^*^p < 0.05, ^**^p < 0.01, and ^***^p < 0.001.

The effect of NANA and NAG on EHEC adhesion was examined by measuring the ability of the EHEC O157:H7 EDL933 strain to adhere to cultured epithelial cells in the presence or absence of NANA or NAG. Our data showed that EDL933 adhesion to HeLa cells was significantly decreased by 40 ± 21 or 23 ± 11% in presence of 1 mM NAG or NANA, respectively (Figure 1A; Figure EV1). At 0.1 mM, only NAG significantly decreased the number of cell-attached bacteria (53 ± 22%).

The adhesion of EHEC O157:H7 to HeLa cells is mainly driven by the production of a T3SS, as previously demonstrated (Branchu et al., 2014) and as verified using the Δ*escN* strain which is defective in the production of the T3SS (Figure EV1). Thus, we investigated the effect of NANA and NAG on LEE gene expression. As a first step, strain EDL933 carrying a P_LEE1_:*lacZ* fusion was cultured in the presence of different concentrations of the sugars. The expression of LEE1 was significantly repressed in the presence of either 1 mM NANA or 0.1 and 1 mM NAG (Figure 1B), but not at 0.01 mM of either sugar. Next, we examined the expression of genes from the five LEE operons and observed that expression of *ler* (LEE1), *sepZ* (LEE2), *escV* (LEE3), *espB* (LEE4), *tir*, and *eae* (LEE5) was significantly lower in presence of 1 mM NANA or NAG (Figure 1C). Consistent with the decreased expression of the LEE4 gene, the secretion of the effector EspB, encoded by the LEE4, was dose dependently reduced when EDL933 was incubated with 0.1 and 1 mM of either sugar (Figure 1D). Taken together, these data indicate that NANA and NAG inhibit the adhesion of EDL933 to epithelial cells and repress T3SS encoding genes. In addition no significant difference was observed upon addition of other mucin sugars, such as mannose, galactose, N-acetylgalactosamine and glucuronate on the expression of *ler* (Figure EV2).

### Repression of LEE gene transcription by NANA and NAG is NagC-dependent

**Figure 2:**
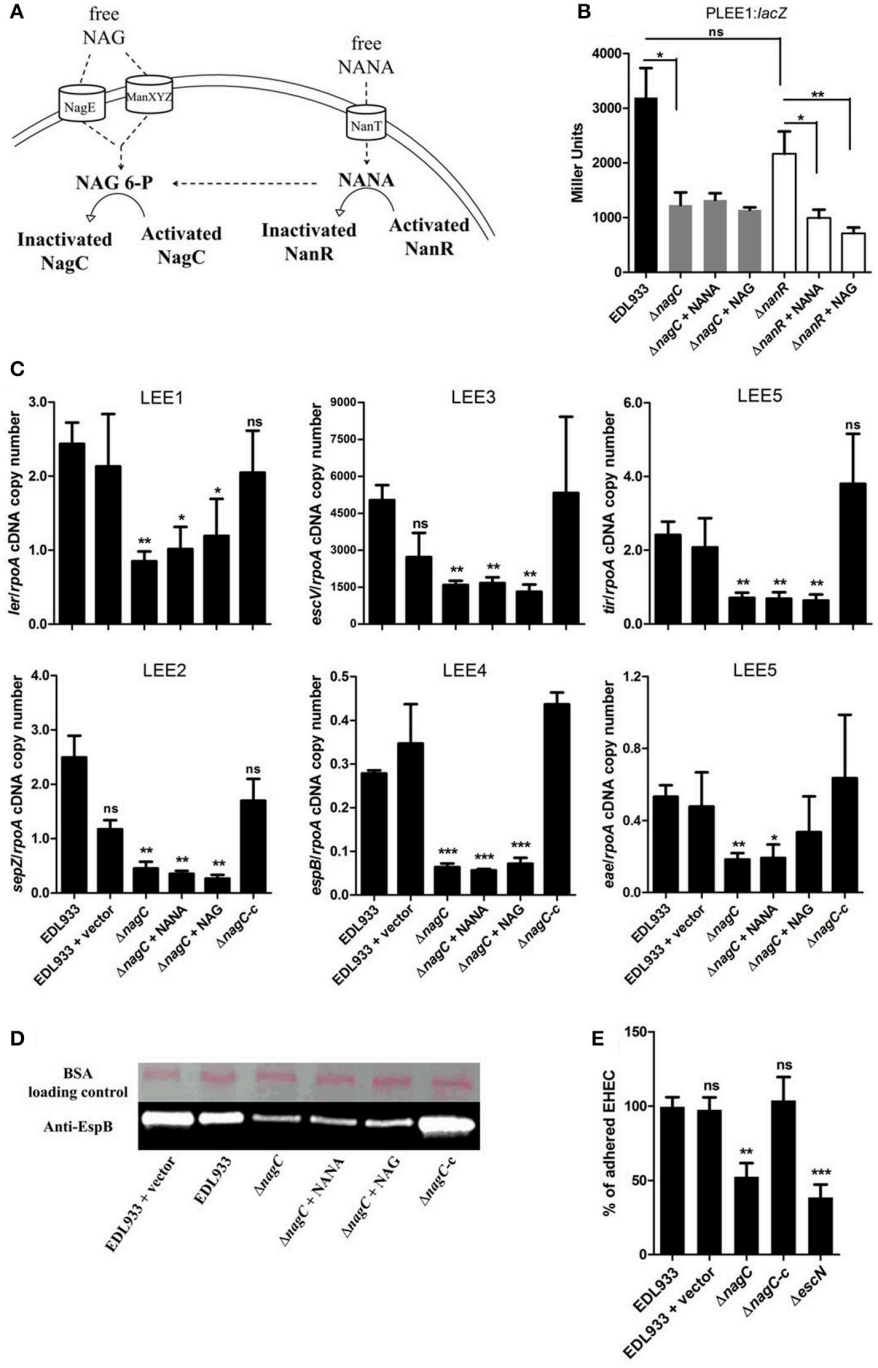
**NagC is a transcriptional activator of the LEE genes. (A)** Schematic representation of the influence of NANA and NAG on the activity of the transcriptional regulators NagC and NanR. **(B)** β-galactosidase assays using the P_LEE1_:*lacZ* transcriptional fusion integrated into EDL933 or the isogenic mutants Δ*nagC* and Δ*nanR*. The strains were grown in DMEM with or without NANA or NAG at 1 mM and harvested at OD_600_ = 0.6. Results are presented as Miller Units. **(C)** qRT-PCR measurement of LEE gene expression. EDL933, the isogenic mutant Δ*nagC* and the complemented strain Δ*nagC*-c were grown in DMEM with or without NANA or NAG at 1 mM. Results are shown as the ratio copy number of the LEE transcripts/copy number of *rpoA* transcripts. **(D)** Western blot analysis of the EspB secretion by EDL933, the isogenic mutant Δ*nagC* and the complement grown in DMEM with or without NANA or NAG at 1 mM. BSA was used as a loading control. **(E)** HeLa cells were co-incubated for 90 min with either the wild type EDL933 strain, Δ*nagC* mutant, the Δ*nagC* complemented strain or the Δ*escN* mutant. Adhered bacteria were harvested and counted on agar plates. Results are presented as the percentage of adhered cells compared to the wild type strain EDL933. *n* ≥ 3, ns for non-significant, ^*^p < 0.05, ^**^p < 0.01, and ^***^p < 0.001.

Activation of the metabolic pathways required for the catabolism of NANA and NAG influence the activity of two transcriptional regulators, NanR and NagC (Figure 2A). Intracellular NANA inactivates NanR while NAG-6P derived from the catabolic conversion of both NANA and NAG inactivates NagC. To evaluate the role of NanR and NagC on LEE1 promoter activity, the P_LEE1_:*lacZ* fusion was introduced into Δ*nanR* and Δ*nagC* mutants. The *nagC* deletion led to a significant decrease of the activity of P_LEE1_ whereas the deletion of *nanR* had no significant effect (Figure 2B). Moreover, the addition of NANA or NAG still repressed the activity of P_LEE1_ in the Δ*nanR* mutant whereas it did not in the Δ*nagC* mutant. These results indicate that LEE1 repression by NANA and NAG was NagC-dependent but NanR-independent. We further demonstrated that a *nagC* deletion also impairs the expression of *escV, sepZ, espB, tir*, and *eae* genes with a fold change ranging from −2.9 to −5.5 (Figure 2C), as well as secretion of EspB (Figure 2D). Consequently, the Δ*nagC* mutant adhered less to epithelial cells, using HeLa and the two human intestinal cell lines HCT-8 and HCT-116 (Figure 2E; Figures EV1, EV3). Importantly, LEE gene expression, EspB secretion and adhesion levels were restored to a wild-type status in a Δ*nagC*-complemented strain. Additionally, we assessed the potential involvement of NagC in the transcription of other genes encoding adhesins in EDL933. Neither *fliC* (flagellin), *ycbQ* (fimbriae) (Samadder et al., 2009), *hcpA* (hemorrhagic coli pilus), *espP* (autotransporter), *lpfA* (long polar fimbriae), *csgA* (curli) nor *pgaB* (poly-β-1,6-N-acetyl-D-glucosamine) were differentially expressed between wild type and Δ*nagC* strains (Supplementary Figure EV4). Since NagC does not affect the synthesis of these adhesins, it suggests that the defect in cell adhesion observed with the *nagC* mutant is mainly driven by a reduced production of the T3SS.

### NagC interacts with LEE1 promoter region *In vitro*

**Figure 3:**
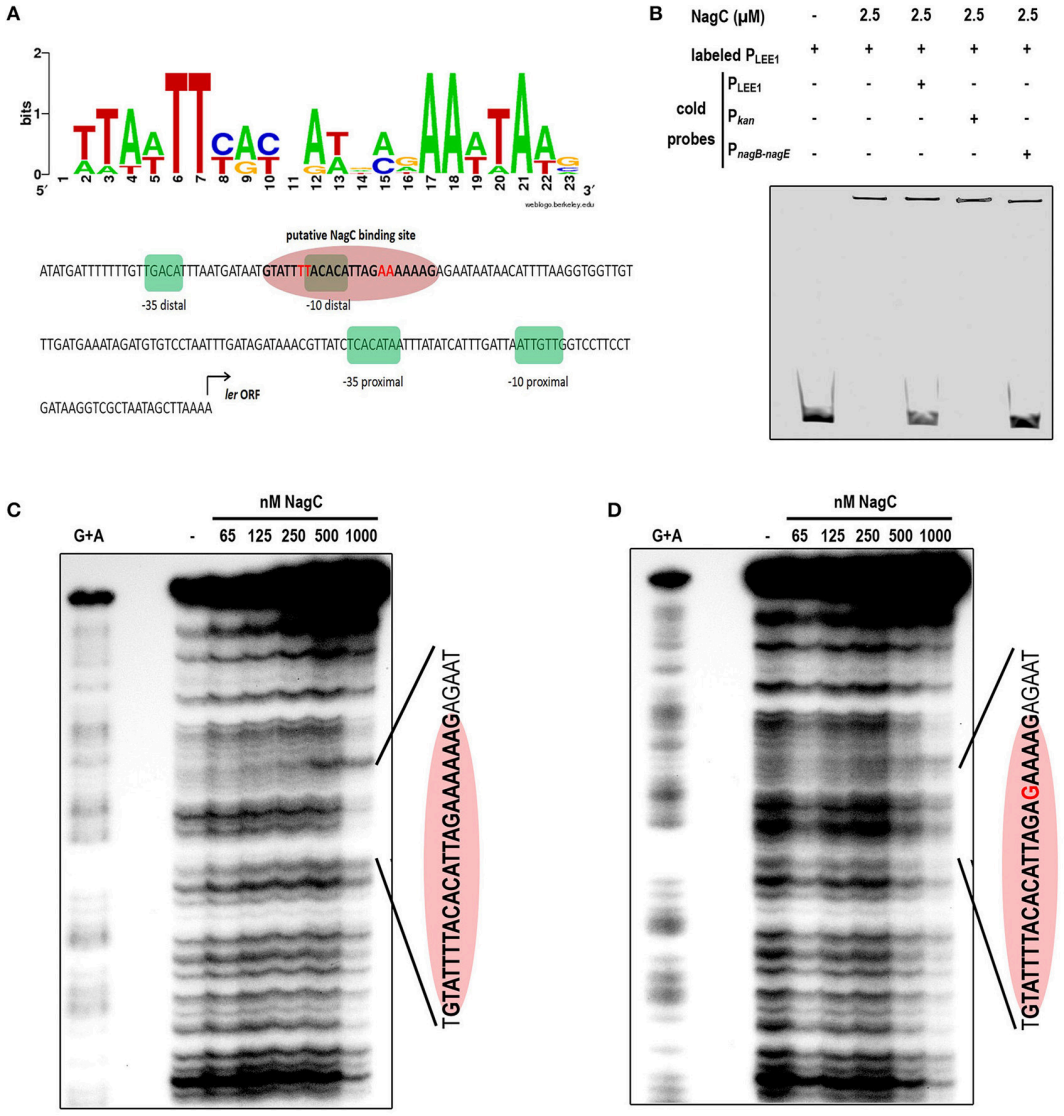
**NagC binds ***in vitro*** to the promoter of LEE1. (A)** Predicted binding site generated by Weblogo from seven known NagC binding sequences and schematic representation of the regulatory region of LEE1. The −35 and −10 boxes of the proximal and the distal promoters are highlighted in green and the putative NagC binding site in red. **(B)** Competitive EMSA assays were performed using purified NagC (2.5 μM) and a 6-FAM labeled P_LEE1_ probe (50 nM) and unlabeled probes corresponding to P_LEE1_, P_kan_ (negative control) or P_*nagB*−*nagE*_ (positive control). **(C)** Footprinting experiment was performed with end-labeled PCR product of the native LEE1 regulatory region and purified NagC. The DNA sequence of the protected region is indicated and includes the NagC putative binding sequence (red ellipse). **(D)** Footprinting experiment was performed with end-labeled PCR product of the mutated LEE1 regulatory region. The base substitution (A → G) is indicated in red.

Using a NagC consensus DNA binding site generated from seven NagC binding sequences, we identified a single putative NagC binding site in the LEE1 promoter region (5′-GTATTTTACACATTAGAAAAAAG-3′) located at a position that overlaps the −10 box of the distal promoter (Figure 3A). The binding of NagC to the LEE1 promoter was first investigated by EMSA that showed that NagC forms a specific low-mobility complex with the LEE1 promoter as previously observed with NagC interacting with type 1 fimbriae *fim* intergenic region upstream of the *fimB* promoter (Sohanpal et al., 2004). Competition EMSA using an unrelated probe (P_kan_) demonstrated that NagC binding to the LEE1 promoter is specific (Figure 3B). Further, DNase footprinting experiments confirmed that NagC bound to the predicted NagC binding site (Figure 3C). Consistent with the expected specificity, a single base substitution at position 18 of the putative NagC binding site prevented DNAse protection by NagC (Figure 3D). An additional DNase footprinting control experiment using *nagBE* intergenic region as expected showed clear protection zones indicating the functional activity of NagC (Figure EV5). These findings demonstrate that NagC interacts with the LEE1 promoter region in a sequence specific manner. Interestingly, the NagC-binding sequence in the promoter of *ler* is conserved among other EHEC O157:H7 strains and this is correlated with ler repression in the presence of 1 mM of NAG (Figure EV6). EHEC, EPEC, or *C. rodentium* strains with degenerated NagC binding site in the LEE1 promoter region were insensitive to NAG exposure.

### Mucin-derived sugars sensing by NagC is important for successful colonization in mice

**Figure 4:**
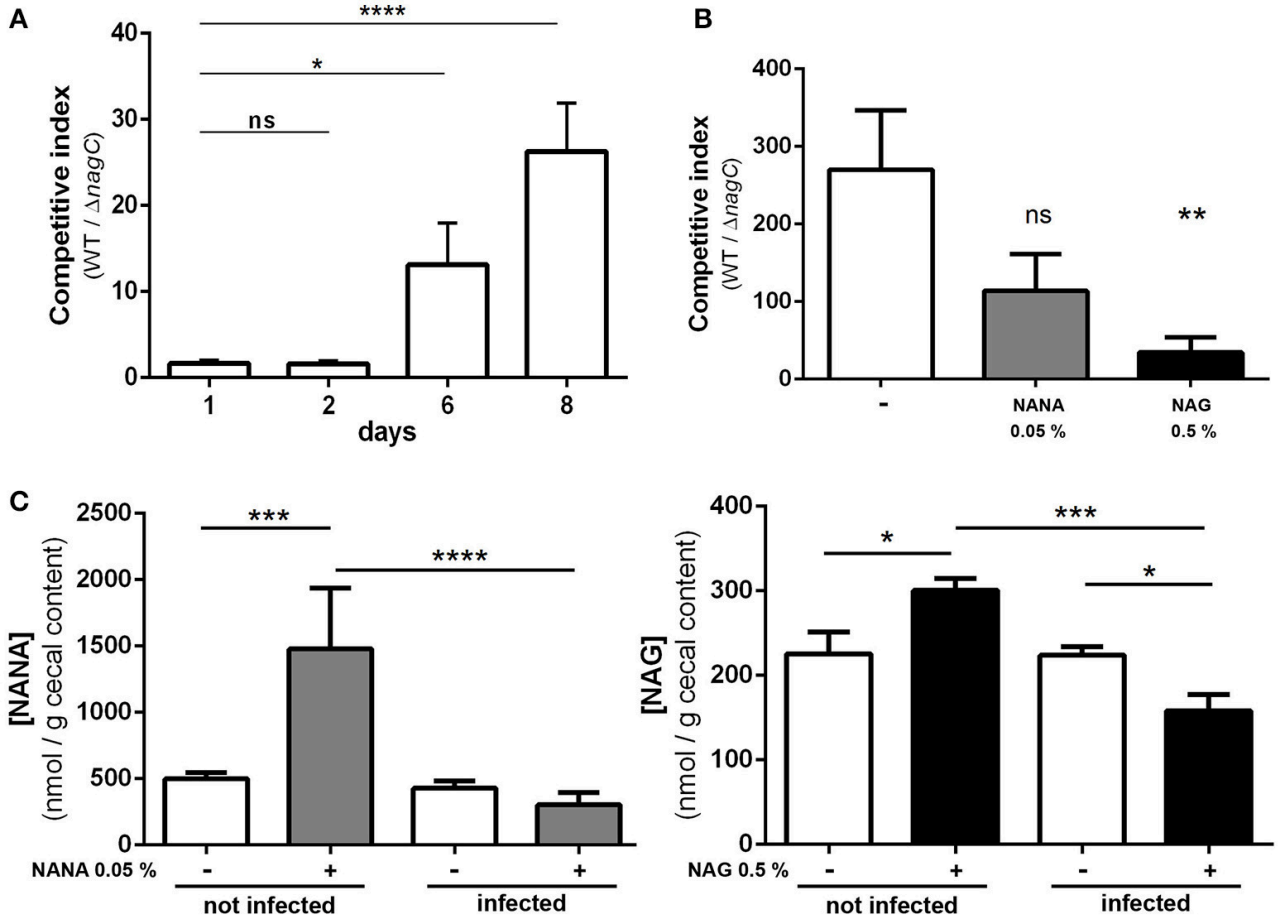
**The Δ***nagC*** mutant is outcompeted by the wild type strain during mice infection**. Streptomycin-treated BALBc mice were infected with a 1:1 mixture of wild type and Δ*nagC* EDL933 strains. **(A)** Wild type and Δ*nagC* strains were numerated from feces and competitive indices WT/Δ*nagC* were calculated at indicated time points. **(B)** Competitive indices WT/Δ*nagC* obtained at day 8 in the cecal contents of mice provided with water with or without NANA 0.05% or NAG 0.5%. **(C)** Concentration of NANA and NAG in the cecal contents of non-infected mice or EHEC-infected mice provided with or without either NANA 0.05% or NAG 0.5%. ^*^p < 0.05, ^**^p < 0.01, ^***^p < 0.001, and ^****^p < 0.0001.

To assess if NagC regulates the gut colonization process, we co-infected mice with an equal mixture of wild-type EDL933 and the Δ*nagC* mutant and followed the outcome of each strain overtime. We observed a marked increase in the wild-type strain over the Δ*nagC* mutant in the feces at days 6 and 8 post-infection (competitive index (CI) of 13 ± 5 and 20 ± 6, respectively), as well as in the cecal content at day 8 (CI of 270 ± 76) (Figures 4A,B). The competitive advantage of the wild-type strain was also recorded for bacteria adhering to cecal and colonic mucosa (Figure EV7). These data demonstrate that the deletion of *nagC* greatly impaired the ability of EDL933 to colonize the intestinal tract of mice.

**Figure 5:**
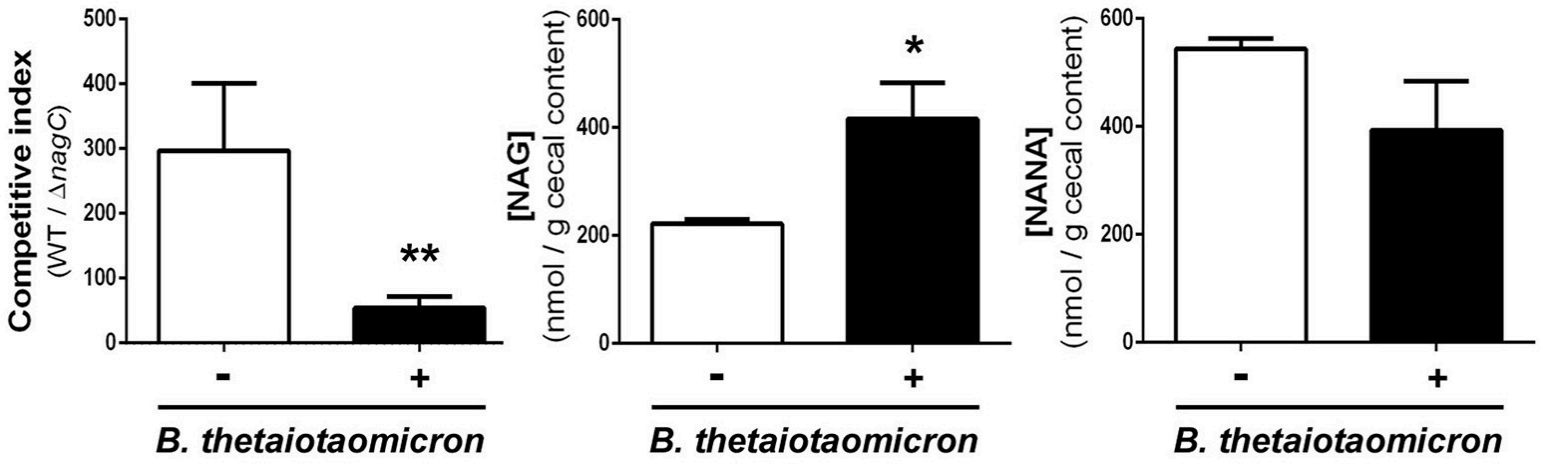
*****Bacteroides thetaiotaomicron*** influences the outcome of wild type and Δ***nagC*** strains in co-infected mice**. Streptomycin-treated BALBc were infected with a 1:1 mixture of wild type and Δ*nagC* EDL933 strains. Mice were gavaged or not daily with 5 × 10^9^ *B. thetaiotaomicron* cells starting 1 day before EHEC infection. Competitive indices WT/Δ*nagC* and concentrations of NAG and NANA obtained at day 8 post-infection in the cecal contents of mice treated or not with *B. thetaiotaomicron* are shown. ^*^p < 0.05 and ^**^p < 0.01.

We next sought to determine if the concentrations of NANA and NAG may alter EDL933 fitness *in vivo* through the modulation of NagC activity. For that, the drinking water of WT/Δ*nagC*-infected mice was supplemented with purified NANA or NAG. Supplementation led to increased sugar concentrations in the cecal content of uninfected mice but not in the cecal content of infected mice (Figure 4C). Interestingly, NANA and NAG concentrations also significantly decreased in supplemented mice upon infection, with fold-change of 4.9- and 1.9, respectively, suggesting that EDL933 consumes NANA and NAG in the intestine of mice. In these conditions, NAG supplementation significantly decreased the competitive advantage of the wild-type strain over the Δ*nagC* mutant by a factor 7.1 (Figure 4B). Co-infected mice were also subjected to a daily gavage with *B. thetaiotaomicron* to see if the behavior of EHEC is modulated by the population level of a mucin degrading bacterium. Gavage of mice did not change NANA concentration in the gut of infected mice but led to a 1.9-fold increase of NAG concentration (Figure 5). Importantly, we observed that the competitive index between the wild-type strain and the Δ*nagC* mutant significantly dropped from 297 in control mice to 54 in *B. thetaiotaomicron*–treated animals (Figure 5). Overall, our findings indicate that NAG concentration in the intestine, derived notably from activity of mucin degrading commensals, such as *B. thetaiotaomicron*, affects the fitness of EHEC *in vivo* in a NagC-dependent manner.

## Discussion

This work demonstrates that the host mucin-derived sugars NAG and NANA inhibit the expression level of LEE genes in EHEC O157:H7 strain EDL933 and, consequently, inhibit the ability of the pathogen to adhere to epithelial cells *in vitro*. NAG and NANA are known to be used as carbon sources by commensal *E. coli* and EHEC O157:H7 in the gut (Fabich et al., 2008; Bertin et al., 2013, 2014; Conway and Cohen, 2015). Their catabolism induces transcriptional responses mediated by NanR and/or NagC regulatory proteins with NanR controlling NANA catabolism and NagC controlling both NAG and galactose catabolism (Plumbridge, 1991; El Qaidi et al., 2009). The role of NagC as a repressor of the expression of *nagE, nagB* and *galP* encoding the NAG PTS permease, the glucosamine-6-P deaminase, and the major galactose transporter respectively, was confirmed in EDL933 (Figure EV8). We also demonstrated that NagC controls the expression level of LEE genes in EDL933 through a direct activation of LEE1 gene transcription. NagC has been shown to modulate the expression of distinct adhesins in other *E. coli* strains. Indeed, NagC, together with NanR, activates the expression of *fimB* encoding for a recombinase required for the expression of the type I fimbriae in K-12 strain MG1655 (McClain et al., 1991; Sohanpal et al., 2004). This is not the case in EDL933 since this strain does not produce type 1 fimbriae due to a 16-bp deletion in the regulatory switch region of *fimA* (Vogeleer et al., 2015). While we observed no change in EDL933, a deletion of *nagC* has been shown to decrease expression of *csgAB* and *csgDEFG* genes and curli production in strain C600, though the mechanism remains unknown (Barnhart et al., 2006). Overall these observations suggest that NagC has served a common role as a regulator of adhesin expression during the evolution of several *E. coli* strains.

Most operons known to be controlled by NagC require two sites for NagC to function (*nagE-B, chb, glmU, fimB*) so that cooperative binding to two sites through DNA looping is necessary for regulation (Plumbridge, 1996; Sohanpal et al., 2004; El Qaidi et al., 2009; Brechemier-Baey et al., 2015). However, like the *galP* promoter, another target of NagC (El Qaidi et al., 2009), only one potential NagC operator is found in LEE1 promoter region of EHEC strain EDL933. Interestingly, this NagC sequence was conserved in other O157:H7 strains but not in other LEE encoding pathogens, such as EPEC E2348/69 or *C. rodentium* ICC168. Yet it is not clear how NagC activates the LEE1 promoter. As proposed by authors working on *fimB* and *galP*, NagC could contact RNA polymerase directly (or another regulatory protein bound closer to the LEE1 promoter region) to enhance transcription activation, or that the nucleoprotein complex that includes NagC and other regulators alters the DNA structure nearer the promoter in such a way as to facilitate transcription initiation (Sohanpal et al., 2004, 2007; El Qaidi and Plumbridge, 2008; El Qaidi et al., 2009).

By regulating genes involved in sugar catabolism and genes involved in T3SS production, NagC is likely to influence the behavior of EHEC during an infection. Indeed, we demonstrated by co-infection experiments that deletion of *nagC* strongly affect the fitness of EHEC in the digestive tract of infected mice. Moreover, the addition of NAG in the drinking water of infected mice reduced the competitive advantage of the wild type strain over the Δ*nagC* mutant. This suggests that intestinal concentration of NAG modulates NagC activation and therefore expression level of NagC-dependent genes, affecting the fitness of EHEC *in vivo*. In the gut, some commensal species expressing mucinolytic enzymes can degrade mucins from the outer layer of mucus and release free carbohydrates into the intestinal lumen (Xu et al., 2003; Elhenawy et al., 2014; Tailford et al., 2015). These sugars can then be consumed by members of the gut microbiota (Derrien et al., 2010). By affecting the concentration of free NAG and NANA available in the digestive tract, gut bacterial species expressing sialidase or N-acetylglucosaminidase might therefore affect the fitness of EHEC through a modulation of NagC activity. Indeed, we showed that the metabolic activity of the mucin degrader *B. thetaiotaomicron* when gavaged to co-infected mice increased the concentration of NAG and reduced the competitive advantage of the wild-type strain over the *nagC* mutant. To our knowledge, no information is available on NAG and NANA concentration in human intestine. However, its concentration probably fluctuates within the gastrointestinal tract since there is high variations in terms of (i) abundance and types of mucins; (ii) patterns of O-glycosylation and (iii) bioavailability of carbohydrates (free or mucin-linked forms). In Figures 4, 5, the concentration of NANA and NAG in the cecal content of uninfected mouse was 0.27 mM (500 nmol/g) and 0.12 mM (225 nmol/g), respectively. In another study, NANA and NAG were quantified in the bovine small intestine content to be 0.1 and 0.45 mM (Bertin et al., 2013). Altogether, it gives information on physiological NAG and NANA concentrations in digestive tracts and indicates that concentrations used in our *in vitro* studies were relevant.

If we determined that NagC is essential for the fitness of EHEC in the digestive tract of mice, we have no information about the NagC-regulated genes involved in fitness alteration. Fabich et al. demonstrated that a mutation in gene *nagE* encoding the NAG transporter, causes a colonization defect for EDL933 in infected mice, indicating that NAG is utilized by the pathogen in the digestive tract of mice. In contrast, a mutation in *nanAT* where *nanT* encodes the NANA transporter, has no impact on colonization efficiency (Fabich et al., 2008), indicating that NANA catabolism is not essential for a good colonization of mice gut. In contrast to *nagE* and *nanAT* mutants that are unable to internalize NAG and NANA respectively, *nagC* mutant is still able to uptake and catabolize both sugars since gene deletion leads to an upregulation of *nagE* and *nagBACD* gene expression (Figure EV8). In addition to sugar catabolism and T3SS related genes, NagC also probably controls the expression of other genes in EHEC, that might be involved in EHEC fitness. One example is the gene z2210 (Figure EV9), which encodes a putative sulfatase that might be involved in mucus degradation since secreted mucins are heavily sulfated (Nieuw Amerongen et al., 1998). NagC of *Vibrio fischeri* was shown to facilitate colonization of the light organ of the squid Euprymna scolopes (Miyashiro et al., 2011). Sun Y et al. proposed that in *V. fischeri* NagC ability to regulate gene expression contributes to its overall fitness in environments that vary in levels of GlcNAc (Sun et al., 2015).

In many pathogens, relationship between metabolism and virulence has been determined (Wilharm and Heider, 2014). We propose that NagC is part of the regulatory circuit controlling the infectious process of EHEC by coordinating mucin-derived sugar metabolism and T3SS production (Figure EV10). At the level of the colonic intestine within the mucus there is a gradient of NANA and NAG due to their release from the intestinal mucin by mucinolytic bacteria, such as *B. thetaiotaomicron* (Derrien et al., 2010). When the concentration of NANA and/or NAG is high, their catabolism by *E. coli* O157:H7 produces high amount of intracellular NAG-6P which inactivates the transcriptional regulator NagC. In such case, the expression of NAG catabolic genes is induced while that of the LEE genes is reduced and thus adherence is prevented. This suggestion is supported by our observation that expression of LEE operons decreased after EDL933 growth in cecal content of gnotobiotic rats inoculated with human cecal content (Le Bihan et al., 2015). In reaching deeper mucus layer toward the intestinal epithelium, NANA and NAG are less found as free forms in the inner layer of mucus but are rather complexed to mucins (Derrien et al., 2010). In consequence, the amount of intracellular NAG-6P is low and the protein NagC is active allowing the repression of *nagB, nagE* and *galP* and activation of the LEE genes and thus adherence is promoted. Such a mechanism could contribute to the relocation of the pathogen from the intestinal lumen to the surface of intestinal epithelial cells, as previously suggested by others (Kamada et al., 2012; Pacheco et al., 2012; Cameron and Sperandio, 2015).

In this study, we described a novel mechanism by which EHEC O157:H7 regulate the expression of its T3SS-encoding genes in response to sugars derived from intestinal mucin. The NAG-6P sensor NagC was shown to promote the adherence of EHEC O157:H7 to intestinal cells *in vitro* through a direct regulation of *ler* and to be an important regulator for the fitness of EHEC *in vivo*. This work sheds further light on the link between the nutrient availability and EHEC O157:H7 adaptation and virulence gene expression.

## Author contributions

Conceived and designed the experiments: GL, CM, JH, and GJ. Performed the experiments: GL, JS, PG, AG, FB, and GJ. Analyzed the data: GL, AB, APG, CM, JH, and GJ. Wrote the paper: GL, CM, JH, and GJ.

## Funding

GL was supported by a scholarship from the Institut de recherche en santé publique de l'Université de Montréal-Fonds de recherche du Québec-santé (Project 40148). This work was also supported in part by the 61st Session de la Commission permanente de coopération franco-québécoise (Project 61. 116 to CM and JH), by the Natural Sciences and Engineering Research Council of Canada (NSERC) (Strategic and Discovery Grants to JH, RGPIN STP 307430 and RGPIN-2015-05373, respectively) and by EADGENE, N FOOD-CT-2004-506416, Network of Excellence under the 6th Research Framework Program of the European Union (CM).

### Conflict of interest statement

The authors declare that the research was conducted in the absence of any commercial or financial relationships that could be construed as a potential conflict of interest.

## References

[B1] Barnhart M. M. Lynem J. Chapman M. R. (2006). GlcNAc-6P levels modulate the expression of Curli fibers by *Escherichia coli*. J. Bacteriol. 188, 5212–5219. 10.1128/JB.00234-06 16816193 PMC1539958

[B2] Bertin Y. Chaucheyras-Durand F. Robbe-Masselot C. Durand A. de la Foye A. Harel J.. (2013). Carbohydrate utilization by enterohaemorrhagic *Escherichia coli* O157:H7 in bovine intestinal content. Environ. Microbiol. 15, 610–622. 10.1111/1462-2920.12019 23126484 PMC3558604

[B3] Bertin Y. Deval C. de la Foye A. Masson L. Gannon V. Harel J.. (2014). The gluconeogenesis pathway is involved in maintenance of enterohaemorrhagic *Escherichia coli* O157:H7 in bovine intestinal content. PLoS ONE 9:e98367. 10.1371/journal.pone.0098367 24887187 PMC4041753

[B4] Branchu P. Matrat S. Vareille M. Garrivier A. Durand A. Crepin S.. (2014). NsrR, GadE, and GadX interplay in repressing expression of the *Escherichia coli* O157:H7 LEE pathogenicity island in response to nitric oxide. PLoS Pathog. 10:e1003874. 10.1371/journal.ppat.1003874 24415940 PMC3887101

[B5] Brechemier-Baey D. Dominguez-Ramirez L. Oberto J. Plumbridge J. (2015). Operator recognition by the ROK transcription factor family members, NagC and Mlc. Nucleic Acids Res. 43, 361–372. 10.1093/nar/gku1265 25452338 PMC4288165

[B6] Cameron E. A. Sperandio V. (2015). Frenemies: signaling and nutritional integration in pathogen-microbiota-host interactions. Cell Host Microb. 18, 275–284. 10.1016/j.chom.2015.08.007 26355214 PMC4567707

[B7] Chekabab S. M. Jubelin G. Dozois C. M. Harel J. (2014). PhoB activates *Escherichia coli* O157:H7 virulence factors in response to inorganic phosphate limitation. PLoS ONE 9:e94285. 10.1371/journal.pone.0094285 24710330 PMC3978041

[B8] Conway T. Cohen P. S. (2015). Commensal and pathogenic *Escherichia coli* metabolism in the gut. Microb. Spectr. 3:MBP-0006-2014. 10.1128/microbiolspec.MBP-0006-2014 26185077 PMC4510460

[B9] Deng W. Puente J. L. Gruenheid S. Li Y. Vallance B. A. Vazquez A.. (2004). Dissecting virulence: systematic and functional analyses of a pathogenicity island. Proc. Natl. Acad. Sci. U.S.A. 101, 3597–3602. 10.1073/pnas.0400326101 14988506 PMC373508

[B10] Derrien M. van Passel M. W. van de Bovenkamp J. H. Schipper R. G. de Vos W. M. Dekker J. (2010). Mucin-bacterial interactions in the human oral cavity and digestive tract. Gut. Microb. 1, 254–268. 10.4161/gmic.1.4.12778 21327032 PMC3023607

[B11] Elhenawy W. Debelyy M. O. Feldman M. F. (2014). Preferential packing of acidic glycosidases and proteases into *Bacteroides* outer membrane vesicles. mBio 5, e00909–14. 10.1128/mBio.00909-14 24618254 PMC3952158

[B12] El Qaidi S. Allemand F. Oberto J. Plumbridge J. (2009). Repression of *galP*, the galactose transporter in *Escherichia coli*, requires the specific regulator of N-acetylglucosamine metabolism. Mol. Microbiol. 71, 146–157. 10.1111/j.1365-2958.2008.06515.x 19007420

[B13] El Qaidi S. Plumbridge J. (2008). Switching control of expression of *ptsG* from the Mlc regulon to the NagC regulon. J. Bacteriol. 190, 4677–4686. 10.1128/JB.00315-08 18469102 PMC2446801

[B14] Fabich A. J. Jones S. A. Chowdhury F. Z. Cernosek A. Anderson A. Smalley D.. (2008). Comparison of carbon nutrition for pathogenic and commensal *Escherichia coli* strains in the mouse intestine. Infect. Immun. 76, 1143–1152. 10.1128/IAI.01386-07 18180286 PMC2258830

[B15] Graveline R. Mourez M. Hancock M. A. Martin C. Boisclair S. Harel J. (2011). Lrp-DNA complex stability determines the level of ON cells in type P fimbriae phase variation. Mol. Microbiol. 81, 1286–1299. 10.1111/j.1365-2958.2011.07761.x 21752106

[B16] Kamada N. Kim Y. G. Sham H. P. Vallance B. A. Puente J. L. Martens E. C.. (2012). Regulated virulence controls the ability of a pathogen to compete with the gut microbiota. Science 336, 1325–1329. 10.1126/science.1222195 22582016 PMC3439148

[B17] Kaper J. B. Nataro J. P. Mobley H. L. (2004). Pathogenic *Escherichia coli*. Nat. Rev. Microbiol. 2, 123–140. 10.1038/nrmicro818 15040260

[B18] Kendall M. M. Gruber C. C. Parker C. T. Sperandio V. (2012). Ethanolamine controls expression of genes encoding components involved in interkingdom signaling and virulence in enterohemorrhagic *Escherichia coli* O157:H7. mBio 3:e00050-12. 10.1128/mBio.00050-12 22589288 PMC3372972

[B19] Le Bihan G. Jubelin G. Garneau P. Bernalier-Donadille A. Martin C. Beaudry F.. (2015). Transcriptome analysis of *Escherichia coli* O157:H7 grown *in vitro* in the sterile-filtrated cecal content of human gut microbiota associated rats reveals an adaptive expression of metabolic and virulence genes. Microb. Infect. 17, 23–33. 10.1016/j.micinf.2014.09.008 25290220

[B20] Leedle J. A. Hespell R. B. (1980). Differential carbohydrate media and anaerobic replica plating techniques in delineating carbohydrate-utilizing subgroups in rumen bacterial populations. Appl. Environ. Microbiol. 39, 709–719. 6769390 10.1128/aem.39.4.709-719.1980 PMC291408

[B21] McClain M. S. Blomfield I. C. Eisenstein B. I. (1991). Roles of *fimB* and *fimE* in site-specific DNA inversion associated with phase variation of type 1 fimbriae in *Escherichia coli*. J. Bacteriol. 173, 5308–5314. 1679430 10.1128/jb.173.17.5308-5314.1991 PMC208240

[B22] McGuckin M. A. Linden S. K. Sutton P. Florin T. H. (2011). Mucin dynamics and enteric pathogens. Nat. Rev. Microbiol. 9, 265–278. 10.1038/nrmicro2538 21407243

[B23] Miller J. H. (1972). Experiments in Molecular Genetics. Cold Spring Harbor, NY: Cold Spring Harbor Laboratory.

[B24] Miyashiro T. Klein W. Oehlert D. Cao X. Schwartzman J. Ruby E. G. (2011). The N-acetyl-D-glucosamine repressor NagC of *Vibrio fischeri* facilitates colonization of Euprymna scolopes. Mol. Microbiol. 82, 894–903. 10.1111/j.1365-2958.2011.07858.x 21992506 PMC3212624

[B25] Nakanishi N. Tashiro K. Kuhara S. Hayashi T. Sugimoto N. Tobe T. (2009). Regulation of virulence by butyrate sensing in enterohaemorrhagic *Escherichia coli*. Microbiology 155, 521–530. 10.1099/mic.0.023499-0 19202100

[B26] Ng K. M. Ferreyra J. A. Higginbottom S. K. Lynch J. B. Kashyap P. C. Gopinath S.. (2013). Microbiota-liberated host sugars facilitate post-antibiotic expansion of enteric pathogens. Nature 502, 96–99. 10.1038/nature12503 23995682 PMC3825626

[B27] Nieuw Amerongen A. V. Bolscher J. G. Bloemena E. Veerman E. C. (1998). Sulfomucins in the human body. Biol. Chem. 379, 1–18. 9504711 10.1515/bchm.1998.379.1.1

[B28] O'Brien A. O. Lively T. A. Chen M. E. Rothman S. W. Formal S. B. (1983). *Escherichia coli* O157:H7 strains associated with haemorrhagic colitis in the United States produce a *Shigella dysenteriae* 1 (SHIGA) like cytotoxin. Lancet 1, 702. 6132054 10.1016/s0140-6736(83)91987-6

[B29] Pacheco A. R. Curtis M. M. Ritchie J. M. Munera D. Waldor M. K. Moreira C. G.. (2012). Fucose sensing regulates bacterial intestinal colonization. Nature 492, 113–117. 10.1038/nature11623 23160491 PMC3518558

[B30] Plumbridge J. (1996). How to achieve constitutive expression of a gene within an inducible operon: the example of the nagC gene of *Escherichia coli*. J. Bacteriol. 178, 2629–2636. 8626331 10.1128/jb.178.9.2629-2636.1996 PMC177988

[B31] Plumbridge J. A. (1991). Repression and induction of the *nag* regulon of *Escherichia coli* K-12: the roles of *nagC* and *nagA* in maintenance of the uninduced state. Mol. Microbiol. 5, 2053–2062. 1766379 10.1111/j.1365-2958.1991.tb00828.x

[B32] Plumbridge J. Kolb A. (1991). CAP and Nag repressor binding to the regulatory regions of the nagE-B and manX genes of *Escherichia coli*. J. Mol. Biol. 217, 661–679. 1848637 10.1016/0022-2836(91)90524-a

[B33] Porter M. E. Mitchell P. Free A. Smith D. G. Gally D. L. (2005). The LEE1 promoters from both enteropathogenic and enterohemorrhagic *Escherichia coli* can be activated by PerC-like proteins from either organism. J. Bacteriol. 187, 458–472. 10.1128/Jb.187.2.458-472.2005 15629917 PMC543544

[B34] Samadder P. Xicohtencatl-Cortes J. Saldana Z. Jordan D. Tarr P. I. Kaper J. B.. (2009). The *Escherichia coli ycbQRST* operon encodes fimbriae with laminin-binding and epithelial cell adherence properties in Shiga-toxigenic *E. coli* O157:H7. Environ. Microbiol. 11, 1815–1826. 10.1111/j.1462-2920.2009.01906.x 19508558 PMC2888687

[B35] Sircili M. P. Walters M. Trabulsi L. R. Sperandio V. (2004). Modulation of enteropathogenic *Escherichia coli* virulence by quorum sensing. Infect. Immun. 72, 2329–2337. 10.1128/IAI.72.4.2329-2337.2004 15039358 PMC375187

[B36] Sohanpal B. K. El-Labany S. Lahooti M. Plumbridge J. A. Blomfield I. C. (2004). Integrated regulatory responses of *fimB* to N-acetylneuraminic (sialic) acid and GlcNAc in *Escherichia coli* K-12. Proc. Natl. Acad. Sci. U.S.A. 101, 16322–16327. 10.1073/pnas.0405821101 15534208 PMC526197

[B37] Sohanpal B. K. Friar S. Roobol J. Plumbridge J. A. Blomfield I. C. (2007). Multiple co-regulatory elements and IHF are necessary for the control of fimB expression in response to sialic acid and N-acetylglucosamine in *Escherichia coli* K-12. Mol. Microbiol. 63, 1223–1236. 10.1111/j.1365-2958.2006.05583.x 17238917

[B38] Sperandio V. Li C. C. Kaper J. B. (2002). Quorum-sensing *Escherichia coli* regulator A: a regulator of the LysR family involved in the regulation of the locus of enterocyte effacement pathogenicity island in enterohemorrhagic *E. coli*. Infect. Immun. 70, 3085–3093. 10.1128/IAI.70.6.3085-3093.2002 12011002 PMC127966

[B39] Sun Y. Verma S. C. Bogale H. Miyashiro T. (2015). NagC represses N-acetyl-glucosamine utilization genes in *Vibrio fischeri* within the light organ of *Euprymna scolopes*. Front. Microbiol. 6:741. 10.3389/fmicb.2015.00741 26236308 PMC4505101

[B40] Tailford L. E. Crost E. H. Kavanaugh D. Juge N. (2015). Mucin glycan foraging in the human gut microbiome. Front. Genet. 6:81. 10.3389/fgene.2015.00081 25852737 PMC4365749

[B41] Vogeleer P. Tremblay Y. D. Jubelin G. Jacques M. Harel J. (2015). Biofilm-forming abilities of Shiga toxin-producing *Escherichia coli* isolates associated with human infections. Appl. Environ. Microbiol. 82, 1448–1458. 10.1128/aem.02983-15 26712549 PMC4771338

[B42] Walters M. Sperandio V. (2006). Autoinducer 3 and epinephrine signaling in the kinetics of locus of enterocyte effacement gene expression in enterohemorrhagic *Escherichia coli*. Infect. Immun. 74, 5445–5455. 10.1128/IAI.00099-06 16988219 PMC1594898

[B43] Wilharm G. Heider C. (2014). Interrelationship between type three secretion system and metabolism in pathogenic bacteria. Front. Cell. Infect. Microbiol. 4:150. 10.3389/fcimb.2014.00150 25386411 PMC4209828

[B44] Xu J. Bjursell M. K. Himrod J. Deng S. Carmichael L. K. Chiang H. C.. (2003). A genomic view of the human-*Bacteroides thetaiotaomicron* symbiosis. Science 299, 2074–2076. 10.1126/science.1080029 12663928

[B45] Yang B. Feng L. Wang F. Wang L. (2015). Enterohemorrhagic *Escherichia coli* senses low biotin status in the large intestine for colonization and infection. Nat. Commun. 6, 6592. 10.1038/ncomms7592 25791315 PMC4382993

