# Supplementary material for: The NAG Sensor NagC Regulates LEE Gene Expression and Contributes to Gut Colonization by Escherichia coli O157:H7
Source: Front Cell Infect Microbiol. 2017 Apr 24;7:134. doi: 10.3389/fcimb.2017.00134 (PMC5401889; doi:10.3389/fcimb.2017.00134)
Supplement: Supplementary file 1 [file Presentation1.PDF]

Supplementary Material Expanded View Figures

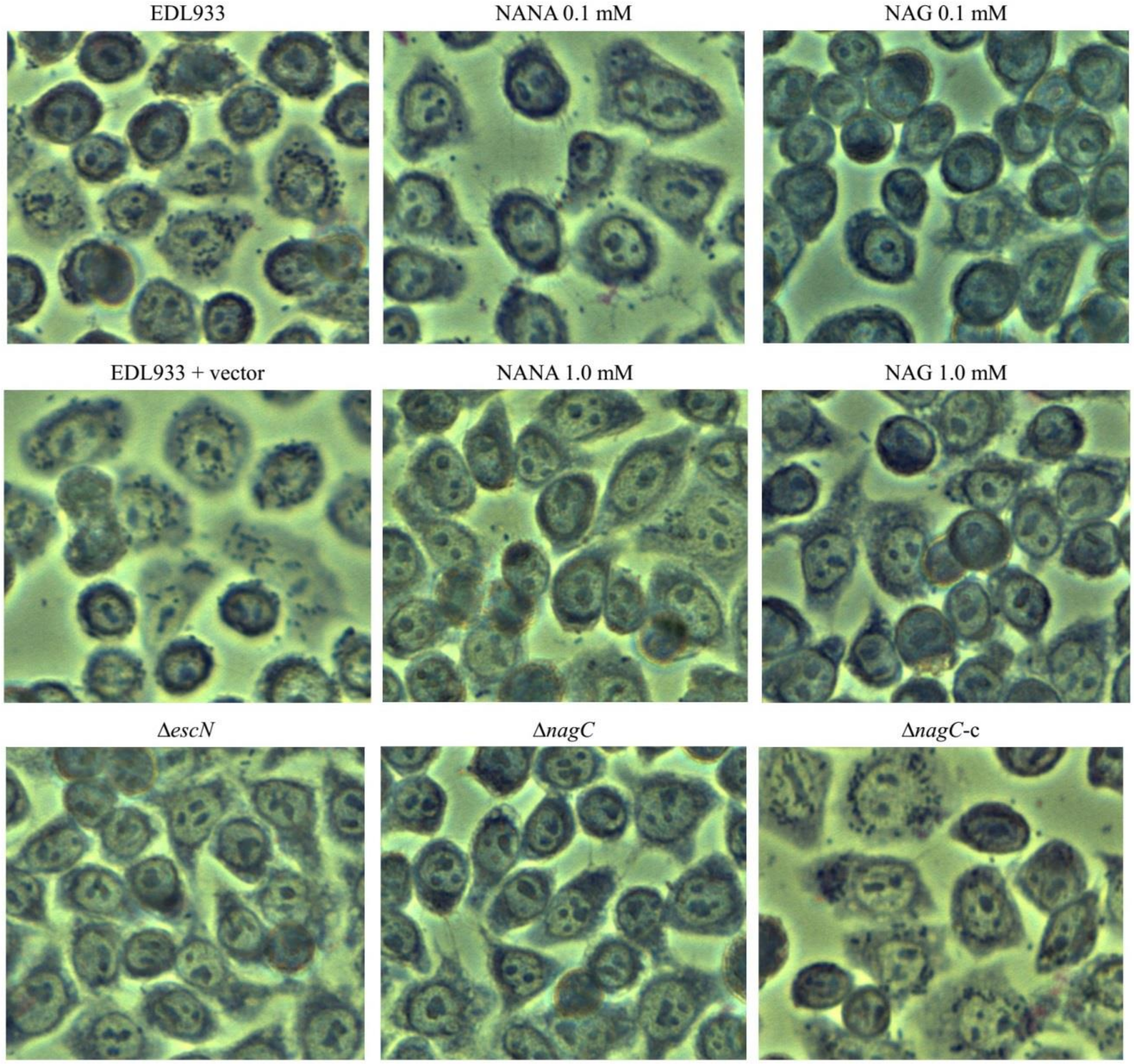

Figure EV1. Adhesion of EDL933, Δ*nagC*, Δ*nagC*-c and Δ*escN* strains to HeLa epithelial cells. Each bacterial strain was co-incubated with HeLa cells for 90 min in presence or absence of NANA or NAG, as described in the material and methods section. Cells were fixed using methanol and stained with Giemsa for 15 min before microscopic visualization. The Δ*escN* strain was used as a negative control for the production of the T3SS.

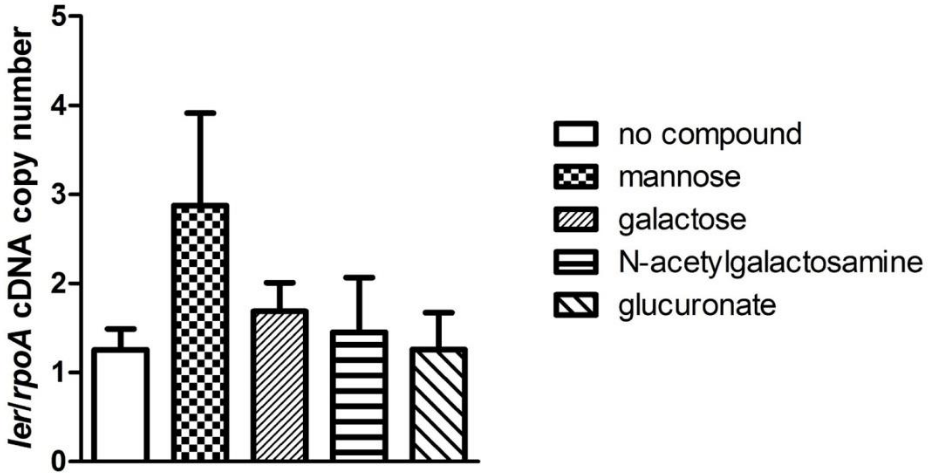

Figure EV2. qRT-PCR measurement of the expression level of *ler* gene in the wild type strain EDL933 after growth in DMEM with or without indicated sugars at concentration of 1mM. Results are shown as the ratio: copy number of the genes transcripts / copy number of *rpoA* transcripts. No significant difference was observed ($p>0.05$)

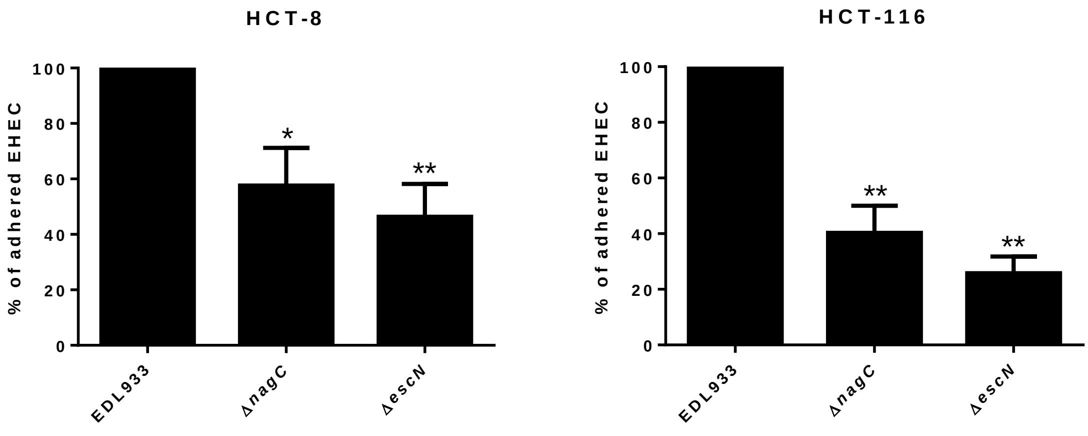

Figure EV3. Adhesion of EDL933 WT and Δ*nagC* mutant to HCT-8 or HCT-116 human colonic epithelial cells. Each bacterial strain was co-incubated with cells for 3 h with an MOI of 10. The number of adherent bacteria was evaluated by CFU counting as described in EV Methods.

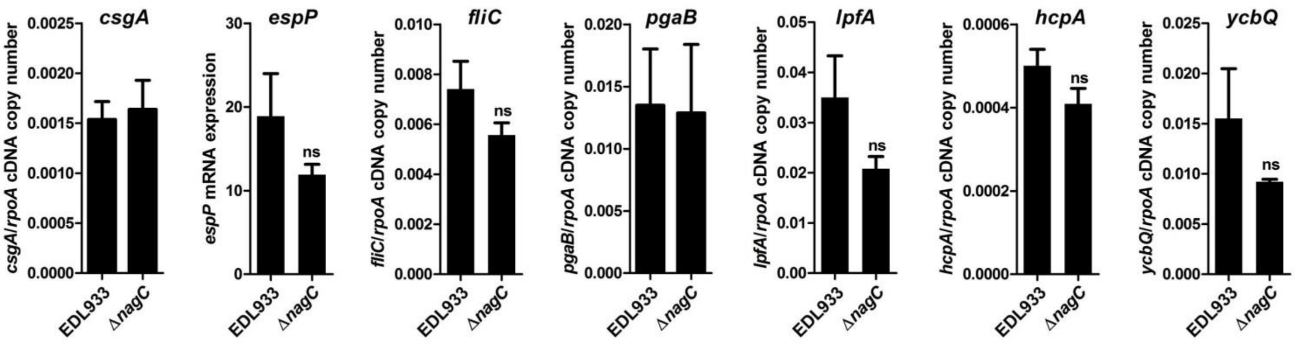

Figure EV4. qRT-PCR measurement of the expression level of the adhesin encoding genes *csgA*, *espP*, *fliC*, *pgaB*, *lpfA*, *hcpA* and *ycbQ* in the wild type strain EDL933 and the Δ*nagC* mutant. Results are shown as the ratio: copy number of the genes transcripts / copy number of *rpoA* transcripts. $n \geq 3$, ns for non-significant.

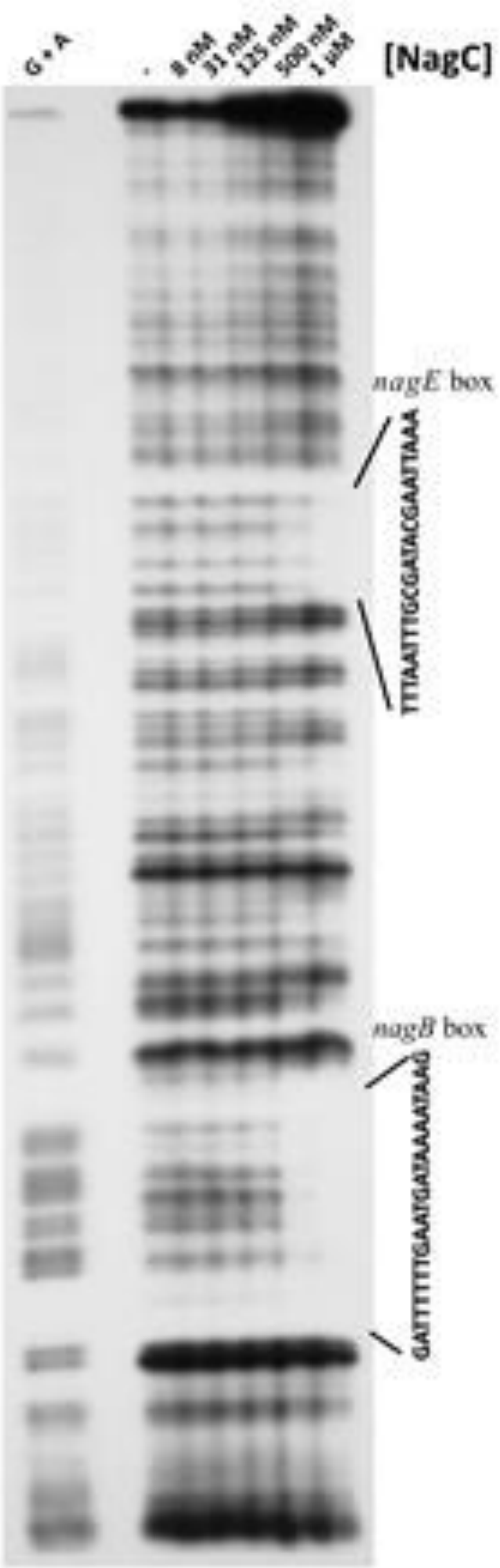

Figure EV5. The Maxam-Gilbert (A+G) reactions and footprinting experiment realized with the forward-labeled PCR product of the original *nagE-nagB* regulatory region and with NagC used at concentrations ranging from to 8 nm to 1 µM. The DNA sequence of the protected region is indicated and the nucleotide sequences corresponding to the NagC putative binding sequences are indicated in bold.

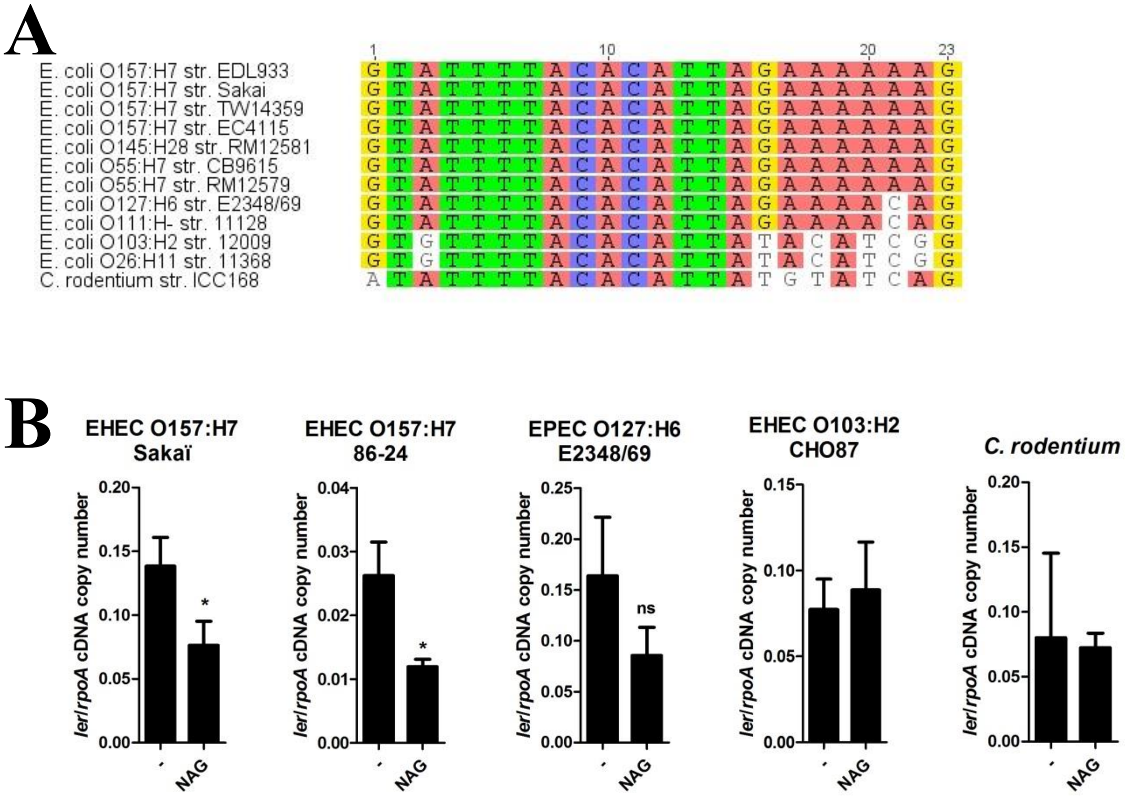

Figure EV6. Conservation of the NagC binding site. (*A*) Alignment of the NagC binding sites located in the promoter of *ler* from selected EHEC, EPEC and *C. rodentium* strains (*B*) qRT-PCR measurement of the expression level of *ler* with or without addition of NAG 1 mM. Results are shown as the ratio: copy number of *ler* / copy number of *rpoA* transcripts. $n \geq 3$, ns for non-significant, * for $p < 0.05$.

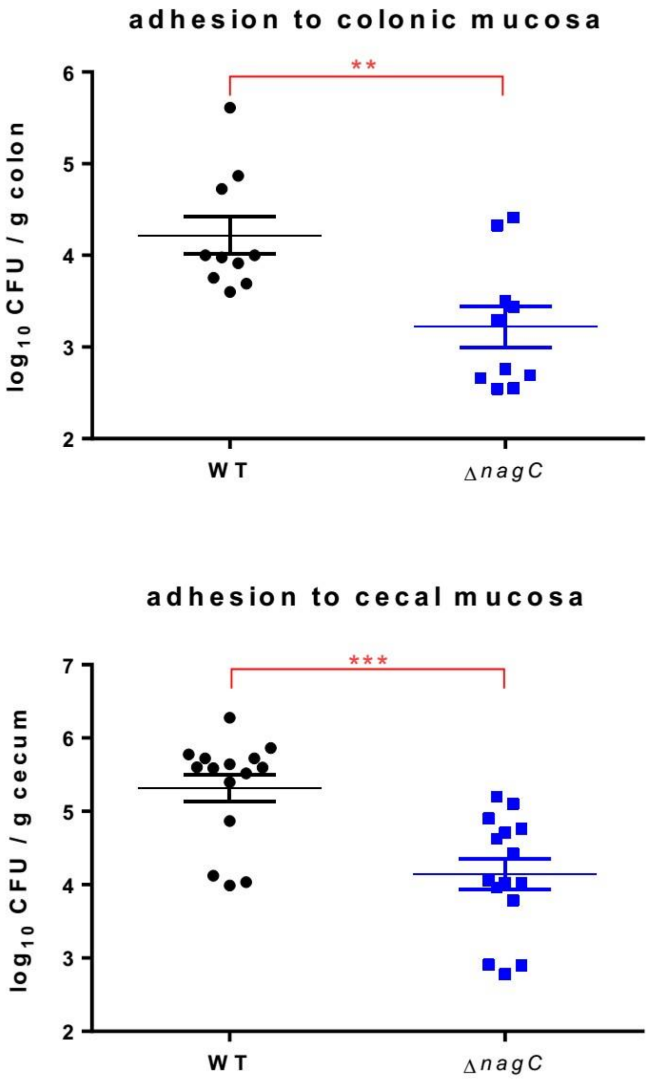

Figure EV7. Adhesion of the wild type strain EDL933 and the Δ*nagC* mutant on the colonic and the cecal mucosa during competition experiments. Streptomycin treated BALBc mice were infected with a 1:1 ratio mixture of WT and Δ*nagC* strains. Eight days after infection, mice were sacrificed and WT (black circle) and Δ*nagC* (blue square) strains were numerated from colonic and cecal tissues. $n \geq 3$, **$p < 0.01$; ***$p < 0.001$.

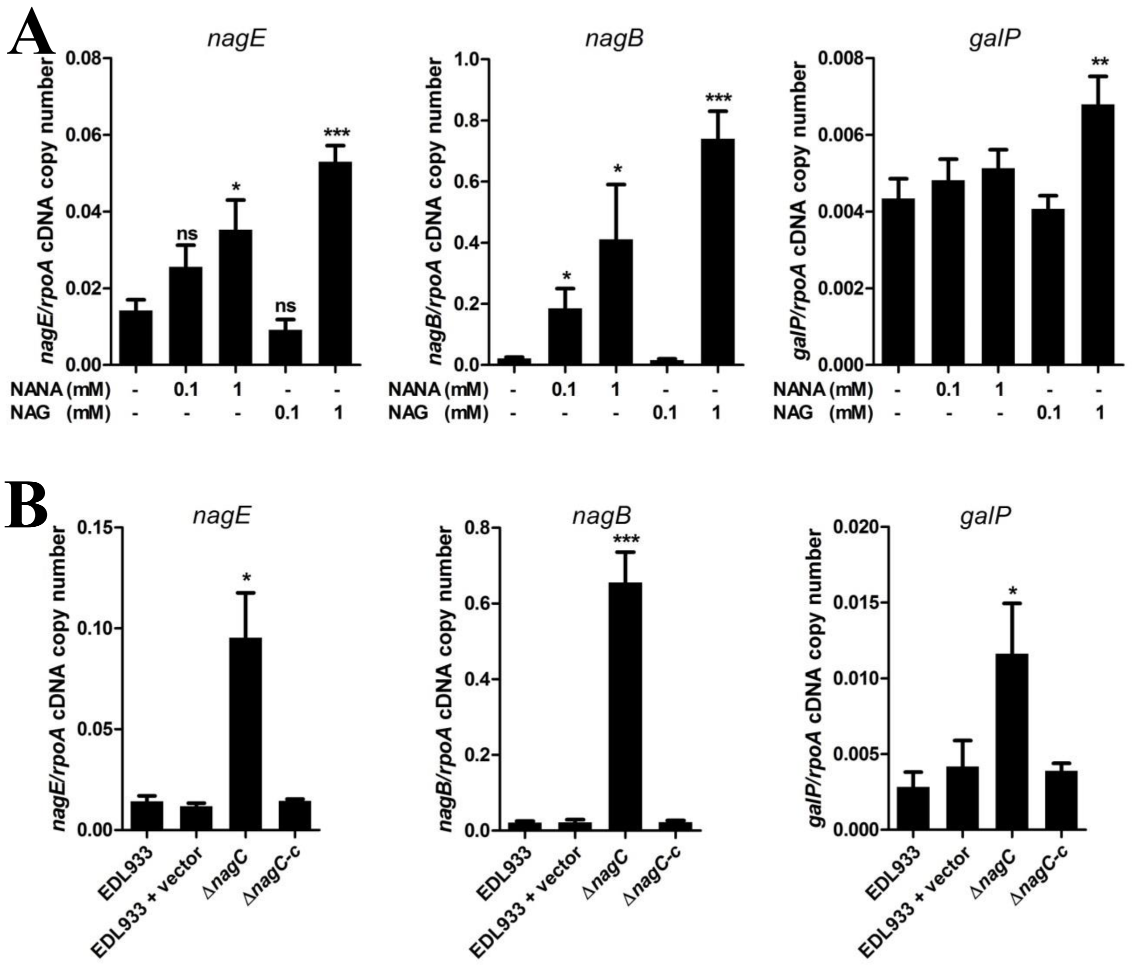

EV8

Figure EV8. qRT-PCR measurement of the expression level of catabolism genes regulated by NagC. (*A*) Effect of the addition of NANA or NAG on the expression level of *nagE*, *nagB* and *galP*. (*B*) Effect of the *nagC* deletion on the expression level of *nagE*, *nagB* and *galP*. Results are shown as the ratio: copy number of the genes transcripts / copy number of *rpoA* transcripts. $n \geq 3$, ns for non-significant, * for $p < 0.05$, ** for $p < 0.01$ and *** for $p < 0.001$.

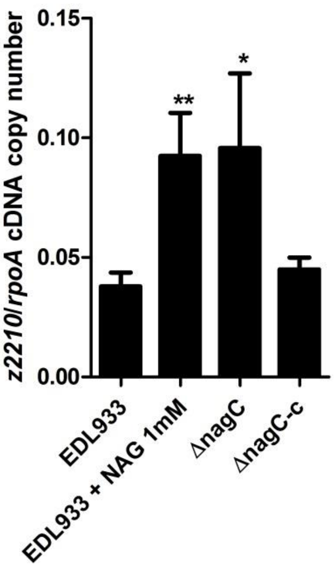

Figure EV9. qRT-PCR measurement of the expression of *z2210* encoding for a putative sulfatase in the wild type strain EDL933 grown with or without NAG 1mM, in the Δ*nagC* mutant and the complemented strain. $n \geq 3$, * for $p < 0.05$ and ** for $p < 0.01$.

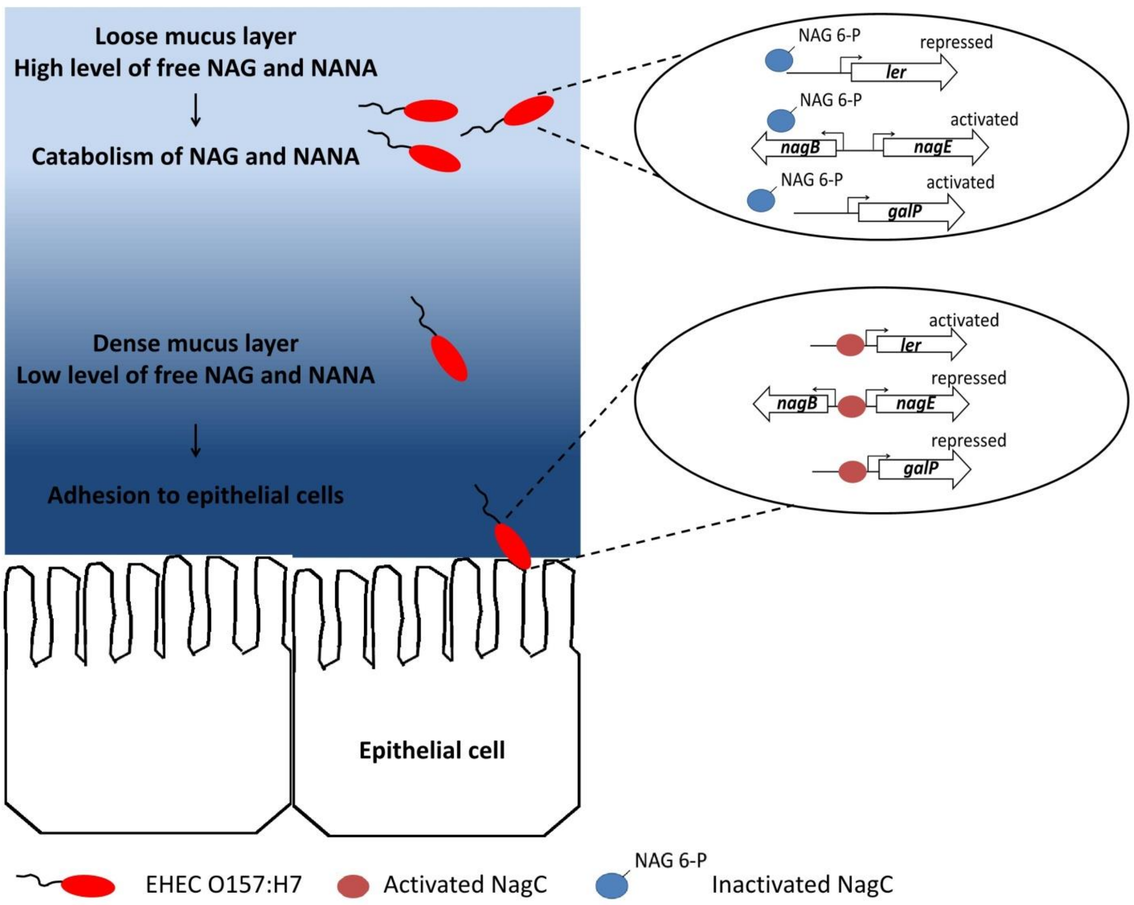

Figure EV10 | Model for the regulation of catabolism and LEE genes by NagC. In our model, at the level of the colonic intestine within the mucus there is a gradient of NANA and or NAG due to the release from the intestinal mucin by glycolytic bacteria of the gut microbiota such as *B. thetaiotaomicron*. When the concentration of NANA and/or NAG is high, their catabolism by *E.coli* O157:H7 produces high amount of intracellular NAG-6P which inactivates the transcriptional regulator NagC. In such case, the expression of *nagB*, *nagE* and *galP* is induced while that of the LEE genes is reduced and thus adherence is prevented. In reaching deeper mucus layer towards the intestinal epithelium, the concentration of NANA and NAG is low, the amount of intracellular NAG-6P is low and the protein NagC is active allowing the repression of *nagB*, *nagE* and *galP* and activation of the LEE genes and thus adherence is promoted.

Expanded View Tables

Table EV1. Bacterial strains and plasmids

**Strains**	**Relevant characteristics**	**References**
EDL933	wild type EHEC O157:H7	(O'Brien et al., 1983)
Δ*nagC*	EDL933 Δ*nagC* KanR	This work
Δ*nanR*	EDL933 Δ*nanR* KanR	This work
Sakai	wild type EHEC O157:H7	(Hayashi et al., 2001)
86-24	wild type EHEC O157:H7	S. Gruenheid, McGill University
E2348/69	wild type EPEC O127:H6	(Elliott et al., 1998)
CHO87	wild type EHEC O103:H2	G. Jubelin, INRA
ICC168	wild type *C. rodentium*	S. Gruenheid, McGill University
χ7213	*thi-1 thr-1 leuB6 glnV44 fhuA21 lacY1 recA1 RP4-2-Tc*::Mu *λpir* Δ*asdA4* Δ*zhf-2*::Tn10	(Roland et al., 1999)
VPI-5482	wild type *B. thetaiotaomicron* strain (ATCC29148) isolated from a healthy adult human	(Xu et al., 2003)
**Plasmids**		
pGEM®-T	Cloning vector	Promega
pMEG-375	Suicide vector *sacRB mobRP4 ori*R6K; CmR ApR	R. Curtiss III, ASU
pTrc99a	expression vector with IPTG inducible *lacI* promoter; ApR	(Amann et al., 1988)
p*nagC*	pTrc99a::*nagC*; ApR	This work
pRS551	*lacZ* reporter gene fusion plasmid; ApR kmR	(Simons et al., 1987)
pGLB	pRS551 containing the intergenic region between *ler* and *espG* (bp-1225 to +19)	This work

Table EV2. List of primers used in this study

Name	Sequence 5' → 3'	Use
tufA-For	GAAGAAGTTGAAATCGTTGG	mRNA quantification
tufA-Rev	GAAGAAGTTGAAATCGTTGG	
rpoA-For	AGCGTATTGCCTACAATGTT	mRNA quantification
rpoA-Rev	CATCACGTAAGTCAACGAAA	
ler-For	AGTTCTACAGCAGGAAGCAA	mRNA quantification
ler-Rev	TTGCGGTAGTAAACACCTTT	
espB-For	AGGTTGCAAATAAAGCTCTG	mRNA quantification
espB-Rev	GCAAGATCTTCAGCAAAGTC	
sepZ-For	ATGGAAGCAGCAAATTTAAG	mRNA quantification
sepZ-Rev	AGCCGTAGTGGTAAGTGCTA	
escV-For	AGAAAACCTTGCAGGTGATA	mRNA quantification
escV-Rev	CCTTATTATTGCCAGTTCCA	
tir-For	TACAGGTCTGATAGGGTTGG	mRNA quantification
tir-Rev	TTTGATTATCTGGGTTCTGG	
eae-For	GTTCTGTCGAATGGTCAAGT	mRNA quantification
eae-Rev	TTGCAGTTCCTGAAACAATA	
gadE-For	AAAATCAATTCCCTGTCAGA	mRNA quantification
gadE-Rev	ATGTGATAAGGTGCCAAAAC	
nanA-For	TATCGGGCAATTATTGATTC	mRNA quantification

nanA-Rev	ATCTGATAGAGATCGCCAGA	
nagE-For	GTACTGGCGGGTATCATTAC	mRNA quantification
nagE-Rev	GCCAGACGTAACCAAAAAT	
nagB-For	CCAATGACCACCTATAAAGC	mRNA quantification
nagB-Rev	CGTTGAGAAGGTTGATGTTT	
galP-For	ACGTACTTGCCACCTTTATC	mRNA quantification
galP-Rev	GACAATAAACATCAGCAGCA	
nagC-For	AATTGGCCATATTCAGGTC	mRNA quantification
nagC-Rev	TGCAGATAGTTTTGATGGTG	
nagD-For	GTTTTATACCTCTGCGATGG	mRNA quantification
nagD-Rev	ATCATGTCCCAGTTGTAGGA	
AscI-nagC-For	AGGCGCGCCTACCGCGCCCGTGGGTGTCCG	Construction of *nagC* mutant
nagC-amont-Rev	TGTGCTTTTATAGTGGCGCTTATTGTTGTC	Construction of *nagC* mutant
H2P2-nagC-For	AGCGCCACTATAAAAGCACAtgtaggctggagctgcttcg	Construction of *nagC* mutant
H1P1-nagC	GTAACTCAATAAGAGAAAGTattccggggatccgtcgacc	Construction of *nagC* mutant
SacI-nagC-Rev	CGAGCTCGGCGATGCCGTATATTACCGG	Construction of *nagC* mutant
nagC-aval-For	ACTTTCTCTTATTGAGTTACGACCTCGTTA	Construction of *nagC* mutant
AscI-nanR-For	AGGCGCGCCTCAATAATTGCCCGATAGTGA	Construction of *nanR* mutant
nanR-amont-Rev	TGCCACTTTAGTGAAGCAGATCGCATTATA	Construction of *nanR* mutant
H2P2-nanR-For	TCTGCTTCACTAAAGTGGCAtgtaggctggagctgcttcg	Construction of *nanR* mutant
H1P1-nanR-Rev	AATCACACTGACCTTACAGAattccggggatccgtcgacc	Construction of *nanR* mutant

SacI-nanR-Rev	CGAGCTCGACCAGACACCGCACATCATC	Construction of *nanR* mutant
nanR-aval-For	TCTGTAAGGTCAGTGTGATTAACATCATCA	Construction of *nanR* mutant
nagC-comp-For-SacI	CGAGCTCGTAACGAGGTCGTAACTCAAT	*nagC* mutant complementation
nagC-comp-Rev-BamHI	CGGGATCCCGGCGCCACTATAAAAGCACAT	*nagC* mutant complementation
P_{LEE1}-For	CGGGATCCATAGCATCATATAGTGTCAA	Plee1-*lacZ* transcriptional fusion
P_{LEE1}-Rev	CGGGATCCGCTTTAATATTTTAAGCTAT	Plee1-*lacZ* transcriptional fusion
P_{LEE1}-mut-For	GTATTTTACACATTAGAGAAAAG	mutagenesis of P_{LEE1}
P_{LEE1}-mut-Rev	CTTTTCTCTAATGTGTAAAATAC	mutagenesis of P_{LEE1}
P_{LEE1}-For 2	TGTTAACGAGATGATTTTCTTCT	footprint and EMSA
P_{LEE1}-Rev	CGGGATCCGCTTTAATATTTTAAGCTAT	footprint and EMSA
C+ Nag For	TGGCGGATTAGGCATCTTTA	footprint and EMSA
C+ Nag	TCCCCCTACGAGAACCCTAT	footprint and EMSA
Reverse 907-F	CGAAACGATCCTCATCCTGT	EMSA (negative control)
Forward 1219-R	AAAGGAAGCGGAACACGTAG	EMSA (negative control)

EV METHODS

STRAIN CONSTRUCTIONS

The EDL933 Δ*nagC* and Δ*nanR* mutants were generated by allelic exchange using a suicide vector as previously in described (Bertrand et al., 2010). Briefly, the kanamycin resistance cassette from pKD13 was amplified and flanked by PCR with the 500 bp sequences adjacent to the ORF of *nagC* or *nanR*, using the primers containing the restriction site for the enzyme *Asc*I or *Sac*I (Table EV2). The resulting amplicon was cloned into the pGEM®-T plasmid (Promega). The amplicon was then removed by enzymatic digestion using *Asc*I and *Sac*I, and ligated into the suicide plasmid pMEG-375. The *λpir* and Δ*asdA4* strain χ7213 was used to transfer the construction into EDL933 by conjugation. A selection of single crossover kanamycin resistant mutants was performed and this was followed by a selection of double crossover mutants using the *sacB* counterselection on LB agar plate containing 5 % sucrose and without NaCl (Kaniga et al., 1991). The EDL933 Δ*nagC* and Δ*nanR* mutants were confirmed by PCR and sequencing. Since *nagC* is in the *nagABCD* operon, the absence of polarity of the *nagC* deletion was verified by analysing the expression of *nagD* by qRT-PCR. Complementation was performed using the pTrc99a expression plasmid. The *nagC* ORF was amplified from the genome of EDL933 using the primers listed in Table EV2 and inserted downstream from the IPTG inducible pTrc promoter into pTrc99a. Experiments using the complemented Δ*nagC* mutant were performed with the basal activity of pTrc, that is, without IPTG induction. The complementation efficiency was verified by quantifying the expression of *nagC* in the wild-type EDL933 and the complemented Δ*nagC* strain via qRT-PCR.

To construct a P_{LEE1}-lacZ fusion, the entire intergenic region between *ler* (LEE1) and *espG* (bp -1225 to +19) containing two *ler* promoters (Porter et al., 2005; Sperandio et al., 2002) was amplified using primers containing a *Bam*HI restriction site (Table EV2) and subcloned into the plasmid pGEM®-T (Promega). The intergenic region was then integrated upstream of the *lacZ* ORF in pRS551 using a *Bam*HI digestion and the T4 ligase (Promega) to yield plasmid pGLB.

Production of The NagC Protein

NagC was produced and purified by Genscript as follows. The NagC gene was cloned in a modified pGEX vector that contained a thrombin protease recognition site between the Glutathione S-Transferase (GST) tag and a multiple cloning site. Following the elution of NagC-GST using Glutathione Sepharose resin, the fused protein was cleaved by thrombin protease to remove the GST affinity Tag. The cleavage reaction was incubated again with Glutathione Sepharose 4B beads and the flow-through containing cleaved NagC was collected. The protein concentration was determined by Bradford protein assay with BSA as a standard, and the purity was about 80 % as estimated by densitometric analysis of the Coomassie Blue-stained SDS-PAGE gel. Functionality of the purified protein was tested by doing a footprinting assay with *nagE-nagB* intergenic region known to be directly regulated by NagC (Fig EV10).

Gel Shift Experiments

Electrophoretic mobility shift assays (EMSA) were carried out with the purified NagC protein and with the Cy5 labeled promoter P_{LEE1}. Briefly, the reaction mix was composed of the purified NagC protein at a concentration of 2.5 µM, the DNA probe at 50 nM and the binding buffer (10 mM Tris pH 8.0, 1 mM EDTA, 1 mM DTT, 5 % glycerol and 10 µg/mL BSA) (Chekabab et al., 2014). When indicated, cold probes corresponding to the promoter of LEE1, *nagB-nagE* or the kanamycin resistance encoding gene from the pKD13 plasmid were added to the mixture at a ratio “cold probe/labeled probe” of 10/1. Reactions were performed at room temperature for 30 minutes and samples were loaded on 4-12 % TBE gels.

Analysis of NagC-Binding Sites

The NagC-binding sequence consensus motif of strain EDL933 was generated from seven NagC-binding sequences located on the promoters of *nagB*, *nagE*, *galP*, *glmU*, *fimB* and *chbB* identified previously (El Qaidi et al., 2009; Plumbridge 1995; Sohanpal et al., 2004) and using the software Weblogo (http://weblogo.berkeley.edu/logo.cgi) as previously described (Branchu et al., 2014).

Mass Spectrometry

The concentrations of NANA and NAG were determined using a High-Performance Liquid Chromatography–Selected Reaction Monitoring Mass Spectrometry (HPLC-SRM MS) assay. Briefly, 50 µL of matrix samples were mixed with 500 µL of internal standard solution (100 µM of stable isotope labeled products of NANA and NAG). The samples were then vortexed vigorously and centrifuged at 12,000 *g* for 10 min and 200 µL of the supernatant was transferred into an injection vial.

Ten µL of samples were injected with a Perkin Elmer Series 200 HPLC (Shelton, CT) onto a Thermo Biobasic C18 5 x 1 mm column (5 µm) with a flow rate of 100 µL/min. The mobile phase consisted of a mixture of methanol and 20 mM ammonium acetate aqueous solution (pH ≈ 7.0) at ratio of 50:50. The AB SCIEX API 2000 QTRAP mass spectrometer (Concord, Ontario, Canada) interfaced with the HPLC system used a pneumatic assisted electrospray ion source operating in negative and positive ion modes. Nitrogen gas 1 was set to 15 PSI; nitrogen gas 2 was set to 25 PSI; and the electrospray electrode was set to -4000 V in negative mode and 5000 V in positive mode. The declustering potential (DP) was set to 30 V and the collision energy (CE) was set to 30 V. The selected reaction monitoring (SRM) transitions were set to 308 → 87 (NANA; negative mode), 311 → 90 ($^{2}H_{3}$-NANA; negative mode), 222 → 204 (NAG; positive mode), 224 → 206 ($^{13}C_{2}$-NAG; positive mode). The dwell time was set to 100 ms and the pause time at 5 ms. Ratios of the light- and heavy-isotopic pairs were used for quantification and Student *t* tests were performed to determine the significance.

Supplementary References

Amann E, Ochs B, Abel KJ. (1988). Tightly regulated *tac* promoter vectors useful for the expression of unfused and fused proteins in *Escherichia coli*. *Gene*. 69, 301-315

Bertrand N, Houle S, LeBihan G, Poirier E, Dozois CM, Harel J. (2010). Increased Pho regulon activation correlates with decreased virulence of an avian pathogenic *Escherichia coli* O78 strain. *Infect. Immun.* 78, 5324-5331. doi:10.1128/IAI.00452-10

Branchu P, Matrat S, Vareille M, Garrivier A, Durand A, Crepin S, Harel J, Jubelin G, Gobert AP. (2014). NsrR, GadE, and GadX interplay in repressing expression of the *Escherichia coli* O157:H7 LEE pathogenicity island in response to nitric oxide. *PLoS Pathog.* 10, e1003874. doi:10.1371/journal.ppat.1003874

Chekabab SM, Jubelin G, Dozois CM, Harel J. (2014). PhoB activates *Escherichia coli* O157:H7 virulence factors in response to inorganic phosphate limitation. *PLoS ONE*. 9, e94285. doi:10.1371/journal.pone.0094285

El Qaidi S, Allemand F, Oberto J, Plumbridge J. (2009). Repression of *galP*, the galactose transporter in *Escherichia coli*, requires the specific regulator of N-acetylglucosamine metabolism. *Mol. Microbiol.* 71, 146-157. doi:10.1111/j.1365-2958.2008.06515.x

Elliott SJ, Wainwright LA, McDaniel TK, Jarvis KG, Deng YK, Lai LC, McNamara BP, Donnenberg MS, Kaper JB. (1998). The complete sequence of the locus of enterocyte effacement (LEE) from enteropathogenic *Escherichia coli* E2348/69. *Mol. Microbiol.* 28, 1-4

Hayashi T, Makino K, Ohnishi M, Kurokawa K, Ishii K, Yokoyama K, Han CG, Ohtsubo E, Nakayama K, Murata T, et al. (2001). Complete genome sequence of enterohemorrhagic *Escherichia coli* O157:H7 and genomic comparison with a laboratory strain K-12. *DNA Res.* 8, 11-22

Kaniga K, Delor I, Cornelis GR. (1991). A wide-host-range suicide vector for improving reverse genetics in gram-negative bacteria: inactivation of the *blaA* gene of *Yersinia enterocolitica*. *Gene*. 109, 137-141

O'Brien AO, Lively TA, Chen ME, Rothman SW, Formal SB. (1983). *Escherichia coli* O157:H7 strains associated with haemorrhagic colitis in the United States produce a *Shigella dysenteriae* 1 (SHIGA) like cytotoxin. *Lancet*. 1, 702

Plumbridge J. (1995). Co-ordinated regulation of amino sugar biosynthesis and degradation: the NagC repressor acts as both an activator and a repressor for the transcription of the *glmUS* operon and requires two separated NagC binding sites. *EMBO J.* 14, 3958-3965

Porter ME, Mitchell P, Free A, Smith DGE, Gally DL. (2005). The LEE1 promoters from both enteropathogenic and enterohemorrhagic *Escherichia coli* can be activated by PerC-like proteins from either organism. *J. Bacteriol.* 187, 458-472. doi:10.1128/Jb.187.2.458-472.2005

Roland K, Curtiss R, 3rd, Sizemore D. (1999). Construction and evaluation of a delta *cya* delta *crp Salmonella typhimurium* strain expressing avian pathogenic *Escherichia coli* O78 LPS as a vaccine to prevent airsacculitis in chickens. *Avian Dis.* 43, 429-441

Simons RW, Houman F, Kleckner N. (1987). Improved single and multicopy lac-based cloning vectors for protein and operon fusions. *Gene*. 53, 85-96

Sohanpal BK, El-Labany S, Lahooti M, Plumbridge JA, Blomfield IC. (2004). Integrated regulatory responses of *fimB* to N-acetylneuraminic (sialic) acid and GlcNAc in *Escherichia coli* K-12. *Proc. Natl. Acad. Sci. U. S. A.* 101, 16322-16327. doi:10.1073/pnas.0405821101

Sperandio V, Li CC, Kaper JB. (2002). Quorum-sensing *Escherichia coli* regulator A: a regulator of the LysR family involved in the regulation of the locus of enterocyte effacement pathogenicity island in enterohemorrhagic *E. coli*. *Infect. Immun.* 70, 3085-3093

Xu J, Bjursell MK, Himrod J, Deng S, Carmichael LK, Chiang HC, Hooper LV, Gordon JI. (2003). A genomic view of the human-*Bacteroides thetaiotaomicron* symbiosis. *Science*. 299, 2074-2076. doi:10.1126/science.1080029
